# Hsp90 induces Acsl4-dependent glioma ferroptosis via dephosphorylating Ser637 at Drp1

**DOI:** 10.1038/s41419-022-04997-1

**Published:** 2022-06-13

**Authors:** Zong Miao, Wei Tian, Yangfan Ye, Wei Gu, Zhongyuan Bao, Lei Xu, Guangchi Sun, Chong Li, Yiming Tu, Honglu Chao, Sin Man Lam, Ning Liu, Jing Ji

**Affiliations:** 1 Department of Neurosurgery, The First Affiliated Hospital of Nanjing Medical University, Nanjing, China; 2 Department of Neurosurgery, Changhai Hospital, Naval Medical University (Second Military Medical University), Shanghai, China; 3 Department of Neurosurgery, The Affiliated Wuxi No.2 People’s Hospital of Nanjing Medical University, Wuxi, China; 4 LipidALL Technologies Company Limited, Changzhou, China; 5 Institute for Brain Tumors, Jiangsu Key Lab of Cancer Biomarkers, Prevention and Treatment, Jiangsu Collaborative Innovation Center for Cancer Personalized Medicine, Nanjing Medical University, Nanjing, China; 6 Gusu School, Nanjing Medical University, Suzhou, China

**Keywords:** Drug development, Drug discovery

## Abstract

Ferroptosis is a newly identified form of regulated cell death (RCD) characterized by the iron-dependent lipid reactive oxygen species (ROS) accumulation, but its mechanism in gliomas remains elusive. Acyl–coenzyme A (CoA) synthetase long-chain family member 4 (Acsl4), a pivotal enzyme in the regulation of lipid biosynthesis, benefits the initiation of ferroptosis, but its role in gliomas needs further clarification. Erastin, a classic inducer of ferroptosis, has recently been found to regulate lipid peroxidation by regulating Acsl4 other than glutathione peroxidase 4 (GPX4) in ferroptosis. In this study, we demonstrated that heat shock protein 90 (Hsp90) and dynamin-related protein 1 (Drp1) actively regulated and stabilized Acsl4 expression in erastin-induced ferroptosis in gliomas. Hsp90 overexpression and calcineurin (CN)–mediated Drp1 dephosphorylation at serine 637 (Ser637) promoted ferroptosis by altering mitochondrial morphology and increasing Acsl4-mediated lipid peroxidation. Importantly, promotion of the Hsp90–Acsl4 pathway augmented anticancer activity of erastin in vitro and in vivo. Our discovery reveals a novel and efficient approach to ferroptosis-mediated glioma therapy.

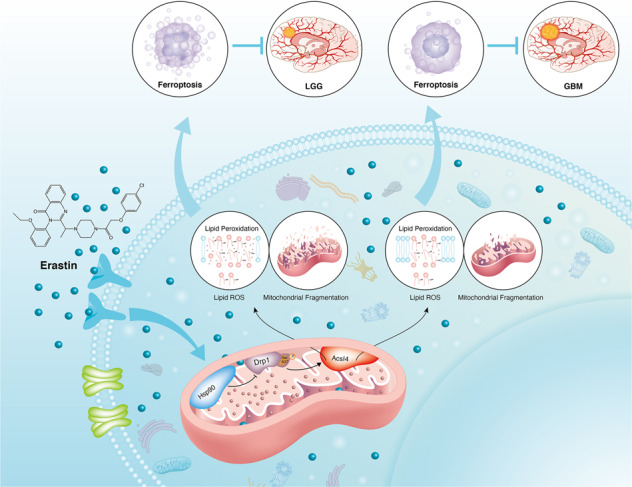

## Introduction

Glioma, the most common type of malignant intracranial tumor, is characterized by aggressiveness and recurrence [1]. Treatment regimens including surgery, radiotherapy, and chemotherapy have been used, based on differing grades of glioma [2]. Despite advances in cancer treatment, patient survival rates and prognoses remain poor due to therapeutic resistance. Glioma therapy requires urgent research into novel therapeutic agents.

Ferroptosis, is a recently recognized form of iron-dependent and Caspase-independent cell death [3]. It differs from other classical types of non-apoptotic cell death, by its biochemical characteristics of iron and lethal-lipid reactive oxygen species (ROS) accumulation, involvement of an individual set of genes, and mitochondrial-morphology shrinkage with condensed mitochondrial-membrane densities [4, 5]. The biochemical mechanism underlying ferroptosis is peroxidation of phospholipids (PLs) that contain polyunsaturated fatty acids (PUFAs), most notably arachidonic acid (AA) and adrenic acid (AdA) [6, 7]. Recent studies have associated ferroptosis with renal failure, intestinal ischemia/reperfusion (I/R), and tumors [8–10]. Additionally, cancer cells that are prone to metastasize or resist to conventional therapies might be vulnerable to ferroptosis [11]. Therefore, it may be promising for cancer therapeutics. Erastin, unlike other ferroptosis inducers such as *Ras*-selective lethal small-molecule 3 (RSL3) and sorafenib, can induce ferroptosis by triggering multiple pathways and has been shown to exert effects in cancer therapy [4, 12].

Acyl–coenzyme A (CoA) synthetase long-chain family member 4 (Acsl4) is a member of the long-chain acyl CoA synthase (ACSL) family, which is imperative in fatty-acid metabolism [13–15]. Recently, Acsl4 has been shown to catalyze the esterification of arachidonoyl and adrenoyl into phosphatidylethanolamine (PE) [16]. Moreover, Acsl4-mediated production of 12- and 15-hydroxyeicosatetraenoic acids (12-HETE, 15-HETE) is essential for ferroptosis [17]. Therefore, Acsl4 is a critical predictor of ferroptosis susceptibility. Hitherto, Acsl4 has rarely been mentioned in gliomas, thus further research is required to understand its role in this malignancy.

In this study, we demonstrated that Acsl4 was highly inducible following erastin treatment of glioma cells. Upregulation of the heat shock protein 90 (Hsp90)–dynamin-related protein 1 (Drp1)–Acsl4 pathway actively regulated ferroptosis via lipid ROS generation and mitochondrial morphology alteration. Mechanically, Hsp90-Drp1 was able to bind and stabilize Acsl4 at protein level. Genetic inhibition of the Hsp90–Drp1–Acsl4 pathway in vitro and in vivo diminished the anticancer activity of erastin-induced ferroptosis. Our findings suggest that Acsl4 could play a unique role in the regulation of ferroptosis and erastin-mediated anticancer therapy. Collectively, we intend to provide a new perspective and target in glioma treatment.

## Methods and materials

### Isolation and culture of cells

Four low-grade glioma (LGG) and seven glioblastoma (GBM) specimens were derived from excess surgical materials of patients (Supplementary. Table S1). All of the patients had signed informed consent and were enrolled in accordance with the institutional protocols (Ethics number:2019-SR-479) approved by the Ethics Committee of the First Affiliated Hospital of Nanjing Medical University. Patient-derived PL1 and PG7 cells were obtained from primary patient brain tumor specimens. In briefly, the divided tumor tissues were digested with 0.1% trypsin (Invitrogen, USA) and DNase I (Promega, USA) for one hour at 37 °C. Erythrocytes were lysed using Red Blood Cell Lysis Buffer (Beyotime, C3702, Shanghai, China). After being washed twice with PBS, the tissues were triturated by pipetting and passed through a 100-μm cell filter. PL1 and PG7 cells were cultured in DMEM containing 10% fetal bovine serum (Gibco, USA) at 37 °C with 5% CO_2_.

### Western blot

After extraction, proteins in cell lysates were first resolved by sodium dodecyl sulfate polyacrylamide gel electrophoresis(SDS-PAGE) and then transferred to nitrocellulose membrane, which was subsequently blocked with 6% nonfat dry milk in TBST for 2 h and incubated with primary antibodies. The primary antibodies used for western blot (WB) analysis were Acsl4 (ab155282), Drp1 (ab184247), Hsp90 (ab59459), MFN1 (ab221661), and MFN2 (ab205236) from Abcam; Calcineurin (CST2614), p-Drp1^Ser637^(CST4867S), and p-Drp1^Ser616^ (CST-3455S) from Cell Signaling Technology; and GAPDH (sc137179) from Invitrogen. After incubation with peroxidase-conjugated secondary antibodies, the signals were probed using the SuperSignal® Maximum Sensitivity Substrate (Thermo Fisher Scientific). The relative band intensity was analyzed using the Image Lab software (Bio-Rad).

### Immunohistochemistry (IHC) assay

Human glioma biopsy specimens and human xenograft mice tumors were fixed with 4% paraformaldehyde, then processed into 10-μm-thick sections and immunostained with specific antibodies for Acsl4, Drp1, p-Drp1^Ser637^,p-Drp1^Ser616^ and Ki67(GB111141). The slides were imaged under a light microscope (Leica, Germany). Percentage of positive cells was calculated by counting under high magnification (×400).

### Lipid ROS imaging and analysis

Lipid ROS imaging: Cells were planted on 6-well chamber slides (5 × 10^5^ cells/well) for 24 h. The slides were washed with PBS and incubated with PBS containing 2 mM BODIPY 581/591 C11 (D3861, Invitrogen) and 200 nM MitoTracker Deep Red FM (Invitrogen) for 20 min. The slides were then imaged using a confocal microscope. (Carl Zeiss Microscopy GmbH, Germany).

Lipid ROS analysis: Lipid ROS was determined using an ROS Assay Kit (Beyotime, S0033S, Shanghai, China) followed by flow cytometry and lipid peroxidation (malondialdehyde, MDA) assay (Beyotime, S0131S, Shanghai, China) in line with the manufacturer’s protocols. Ferrostatin-1 (S7243) and ZVAD-FMK (S7023) were purchased from Selleck Chemicals.

12-HETE and 15-HETE levels were detected respectively by 12-HETE ELISA kits (ab133034, Abcam) and 15-HETE ELISA kits (ab133035, Abcam) according to the manufacturer’s instructions.

### GSH and GPX activity assay

Reduced glutathione (GSH) was detected by GSH and GSSG Assay Kits (Beyotime, S0053, Shanghai, China). Glutathione peroxidase (GPX) activity was determined using a Glutathione Peroxidase Assay Kit (Beyotime, S0056, Shanghai, China).

### Co-immunoprecipitation and silver staining

PL1 cells were washed with pre-cold PBS and then lysed using RIPA Lysis Buffer (Beyotime, P0013D, Shanghai, China) containing protease inhibitors (PMSF). The lysate supernatants were incubated with Acsl4 antibody (ab155282) or IgG antibody (Beyotime, A0192, Shanghai, China) overnight at 4 °C. The immunocomplex precipitations were formed when protein A/G plus agarose (sc2003) was added at 4 °C for another 6 h. Microbeads were washed with 1x loading buffer to remove unbound proteins. Precipitated proteins were analyzed by immunoblotting. Mouse IgG was used as negative control. Silver staining was performed using a Fast Silver Stain Kit (Beyotime, P0017S, Shanghai, China) in accordance with the manufacturer’s protocol.

### Immunofluorescence staining

Cells were planted on 24-well plates chamber slides and grew overnight to adhere. Dual immunostaining was performed sequentially. First, the cells were fixed with cooled 4% paraformaldehyde for 30 min and permeabilized with 0.25% Triton X-100 for 1 h. After being blocked with 5% BSA for 1 h, the cells were incubated in 5% BSA at 4 °C overnight with the primary antibody. Next, the cells were washed with PBS twice and incubated in 5% BSA for 1 h at room temperature with secondary antibodies Alexa Fluor 488 (Lot:12194) and Cy3(Lot:125099) from Jackson ImmunoResearch (USA). Nuclei were stained with Hoechst (Beyotime, 33342, Shanghai, China). Photographs were taken using a confocal microscope (Carl Zeiss Microscopy GmbH, Germany).

### Flow cytometry

Cells were digested and washed twice with PBS, centrifuged at 2000 rpm for 5 min and aliquoted 3 × 10^5^ cells in flow cytometry tubes. The cells were fixed using cold 4% paraformaldehyde for 10 min, permeabilized with 0.25% Triton X-100 for 15 min and blocked for 30 min at room temperature with 5% BSA. They were then immunostained in 0.1% Triton X-100 and 1% BSA at 4 °C for one hour using p-Drp1^Ser637^ (CST4867S) and p-Drp1^Ser616^ (CST-3455S) and secondary antibody Alexa Fluor 488 (Lot:12194). Labeled cells were resuspended in 300 µL PBS and analyzed using BD LSR Fortessa™ X-2ab150077ll analyzer (BD Biosciences, San Jose, CA). Data were analyzed using the FlowJo -V10.

### Cellular thermal shift assay

PL1 and PG7 cells of the indicated groups were treated with erastin of different concentrations for 6 h. The cells were harvested and washed with PBS, resuspended to a density of 5 × 10^6^ cells/mL in PBS added with protease inhibitor, and then lysed by three cycles of flash-freeze-thawing using liquid nitrogen and 23 °C water. The cell lysates were collected after centrifugation at 15000 rpm for 15 min at 4 °C. Then, the supernatants were analyzed with WB assay.

### Mitochondrial membrane potential (MMP) assay

The mitochondrial membrane potential was determined using the JC-1 Assay Kit (Beyotime, C2003S, China), and then quantified by flow cytometry analysis.

### Colony formation assay

Cells were planted in culture dishes (1 × 10^6^ cells/dish) and cultured for two weeks. Subsequently, they were washed with PBS, fixed with 4% formaldehyde for 10 min, and stained with 0.5% crystal violet for 30 min. Colonies containing >50 stained cells were classified as clones.

### Cell viability analysis and lactate dehydrogenase (LDH) assay

Cells were seeded in a 96-well plate, and cell viability was determined by Cell Counting Kit-8(CCK-8) assay (Beyotime, C0037, Shanghai, China) in accordance with the manufacturer’s instructions. The absorption was measured at 450 nm with a microplate reader. LDH release was measured using an LDH Cytotoxicity Assay Kit (Beyotime, C0016, Shanghai, China) in accordance with the manufacturer’s instructions. The absorbance was then measured at 490 nm with the microplate reader.

### Vectors and lentiviral transfection

The lentivirus-based plasmid shRNA of PL1 targeting Acsl4 and Hsp90, and the overexpression plasmid vector of PG7 targeting Acsl4 and Hsp90 were purchased from Genepharma (shAcsl4 target sequence: 5′-AUUGCUAUGAUGCAUCAUCAC UCCC3′; shHsp90 target sequence: 5′-CCAACTCATGTCCCTCATCAT-3′; shMFN1 target sequence: 5′-AAGGGGAUUACUGCAAUCUUU-3′; shMFN2 target sequence: 5′-AAGAGACACAUGGCUGAGGUG-3′). Acsl4-overexpression vectors: forward 5′-TTTAAACTTAAGCTTGGTACCATGGCAAAGAGAATAAAAGCTAAGC-3′ and reverse 5′-AACGGGCCCTCTAGACTCGAGTTATTTGCCCCCATACATCCG-3′; Hsp90 overexpression vectors: forward 5′-AGTCTCGAGGTCACCAGAACTA TGTGTTTG and reverse 5′-ATTGCGGCCGCATCTCCTCTGTATTTATCT. The plasmids were transfected into the cells using Lipofectamine 2000 (Invitrogen, Carlsbad, CA, USA) in accordance with the manufacturer’s protocol. Drp1^S637E^ and Drp1^S637A^ mutants were obtained using the QuickChange Multi III Site-Directed Mutagenesis Kit (Stratagene, North Torrey Pines, CA) and verified by sequencing. Primer sequences were as follows: Drp1^S637E^ forward: ATTCCAATTATGCCAGCCG AGCCACAAAAAGGTCATGCCGT and reverse: ACGGCATGACCTTTTTGTGG CTCGGCTGGCATAATTGGAAT; Drp1^S637A^ forward: GTTCCTGTTGCACGAAA ACTAGCTGCTCGGGAAC and reverse: GTTCCCGAGCAGCTAGTTTTCGTGCA ACAGGAAC.

### Quantitative RT-PCR

Total RNA was extracted from PL1 and PG7 cells using TRIzol reagent (Thermo Fisher Scientific, USA) following the manufacturer’s instructions. Primer sequences were as follows: Acsl4 forward: 5′-GCTACTTGCCTTTGGCTCATGTGC-3′ and reverse: 5′-GTGTGGGCTTCAGTACAGTACAGTCTCC-3′; MFN1 forward: 5′-GGCATCTGTGGCCGAGTT-3′ and reverse: 5′- ATTATGCTAAGTCTCCGCTCCAA-3′; MFN2 forward: 5′-CTGCTAAGGAGGTGCTCAA-3′ and reverse: 5′-TCCTCACTTGAAAGCCTTCTGC-3′; GAPDH forward: 5′- GTCTCCTCTGACTTCAACAGCG-3 ′ and reverse: 5′-ACCACCCTGTTGCTGTAGCCAA-3′.

### 5-ethynyl-2'-deoxyuridine (EdU) assay

Cell proliferation was determined using an EdU Proliferation Kit (Beyotime, C0071S, Shanghai, China). Cells were cultured in a 48-well plate for 24 h, then incubated with 50 mM EdU solution for 2 h and fixed in 4% paraformaldehyde. Subsequently, the cells were permeabilized with 0.25% Triton X-100 for 15 min and sequentially stained with Alexa Fluor 488 (Lot:12194) and Hoechst (Beyotime, 33342, Shanghai, China).The EdU-treated cells were then imaged and assessed using an Olympus FSX100 microscope (Olympus, Tokyo, Japan).

### Terminal deoxynucleotidyl transferase dUTP nick end labeling (TUNEL) assay

PL1 cells, PG7 cells, and xenograft tumor sections were fixed in 4% paraformaldehyde for 15 min. TUNEL staining was performed with a One-Step TUNEL Apoptosis Assay Kit (Beyotime, C1086, Shanghai, China) according to the manufacturer’s protocol. Images were acquired with an Olympus FSX100 microscope (Olympus, Tokyo, Japan).

### Lipidomics analysis

Lipidomics analysis was performed by LipidALL Technologies Co., Ltd. (Changzhou, China). Lipids were extracted from approximately 20 mg of tissues or 1 × 10^6^ cells using the method described previously [18].

### Mass spectrometry

Liquid chromatography (LC) with tandem mass spectrometry (MS) was carried out by BGI Tech Solutions Co., Ltd (BGI Shenzhen, Guangdong, China). Protein pellets were digested with trypsin to a protein ratio of 1:20 and incubated at 37 °C for 4 h. For each sample, the equivalent of 2–5 mg of protein was loaded into the LC-MS/MS.

### Xenograft mouse model

The six-week-old male nude mice used in this study were purchased from Nanjing Medical University Animal Center. For intracranial GBM xenograft experiments, PG7 cells lentivirally transduced with firefly luciferase (Fluc) were implanted into the frontal subdural region. The IVIS Imaging System (Caliper Life Sciences) was used to measure intracranial tumor growth. Each mouse was intraperitoneally injected with 10 mg D-luciferin (YEASEN, Shanghai, China) before imaging. The Living Images software package (Caliper Life Sciences) was used to analyze the integrated flux of photons in each region. The procedures were approved by the Animal Management Rule of the Chinese Ministry of Health (documentation 55, 2001) and the Nanjing Medical University Animal Experimental Ethics Committee (Ethics number: IACUC-1907006).

### Statistical analyses

Statistical analyses were performed using the Prism 8.0.2 software (GraphPad Software, USA). Quantitative data were compared using a Student’s *t*-test between two samples or one-way analysis of variance (ANOVA) for multiple samples. Statistical significance with the Kaplan–Meier survival curves was calculated with the log-rank test. Data of subcutaneous tumor diameters in each group were analyzed using a two-way ANOVA. All results were indicated as the mean ± S.D. and repeated in at least three independent experiments. *P*-value < 0.05 was considered statistically significant.

## Results

### Acsl4 contributed to lipidomic differences in gliomas

**Fig. 1:**
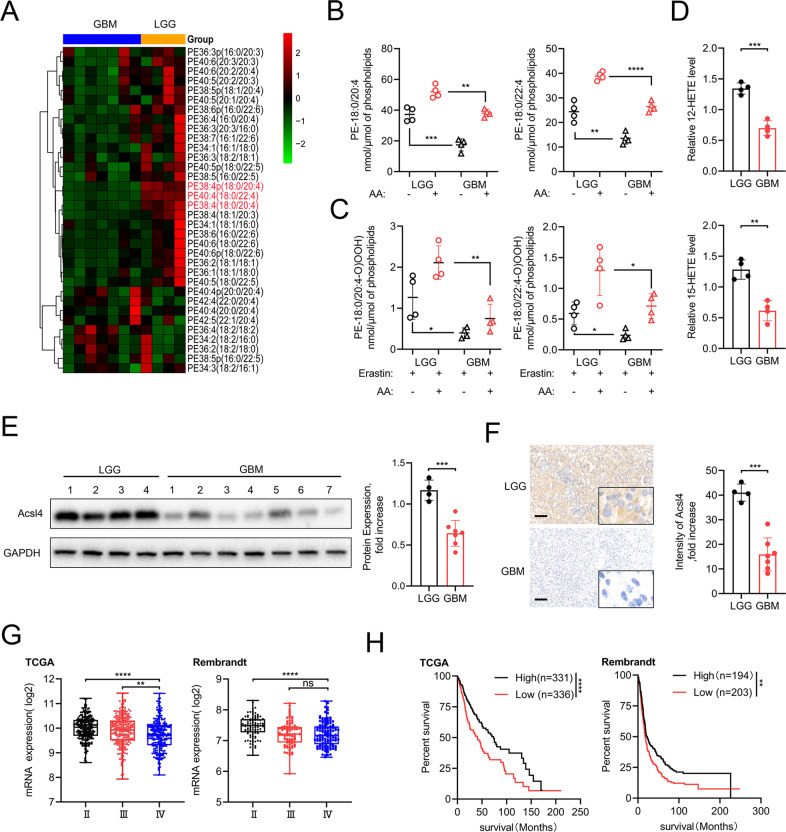
Acsl4 contributs to lipidomic difference in gliomas. **A** Heat map of all major PE species was classified into LGG and GBM clusters. **B** Quantitative analysis of PE (18:0/20:4) and PE (18:0/22:4) in LGG and GBM cells in the absence or presence of AA. The cells were supplemented with AA (3.5 μM, 16 h at 37 °C). Data indicated as mean ± S.D. (*n* = 4 experiments). **C** Quantitative analysis of hydroperoxy-PE (18:0/20:4) and hydroperoxy-PE (18:0/22:4) in LGG and GBM cells in the absence or presence of AA. The cells were supplemented with AA (3.5 μM, 16 h at 37 °C) and treated with erastin (5 μM, 6 h at 37 °C). **D** 12-HETE and 15-HETE levels were detected in LGG and GBM cells. Data indicated as mean ± S.D. (*n* = 4 experiments). **E**, **F** Acsl4 protein in human glioma (LGG, *n* = 4; GBM, *n* = 7) samples was evaluated by western blot and IHC. GAPDH was used as control in western blot assays. **G** Database analysis (TCGA, Rembrandt) of different grades of human primary gliomas. Expression of Acsl4 mRNA in LGG (WHO II) was compared to that of GBM (WHO IV). **H** Kaplan–Meier survival analysis (TCGA, Rembrandt) of high versus low Acsl4-expressing gliomas. Log-rank test. Scale bars: 100 μm. *p < 0.05, **p < 0.01, ***p < 0.001.

To investigate the underlying roles of ferroptosis in gliomas, microarray assays were first performed to identify lipid oxidation events by analyzing all major PE species in LGG and GBM specimens. We found that AA- and AdA-containing PE (18:0/20:4 and 18:0/22:4, respectively) species were strikingly reduced in GBM *versus* LGG tissues (Fig. 1A) and cells (Fig. 1B), so did the formation of doubly and triply oxidized AA- and AdA-containing PE species, 12-HETE and 15-HETE levels (Fig. 1C, D).

Previous research demonstrated that several encoding proteins are essential for lipid biosynthesis, specifically in catalyzing PE-AA and PE-AdA, which are preferred oxidation substrates [15]. Next, we detected endogenous levels of four proteins—Acsl4, GPX4, LPCAT3, and 15-LOX in LGG and GBM specimens using WB. Acsl4 was selected for further analysis because other proteins showed no difference in expression between LGGs and GBMs (Fig. 1E, F, Supplementary Fig. 1A). Then, analysis of Acsl4 expression in public databases such as The Cancer Gene Atlas (TCGA) and Rembrandt revealed its relatively low expression in GBM compared with LGG (Fig. 1G). The Kaplan–Meier analysis also demonstrated that patients with low Acsl4 expression levels displayed reduced overall survival time (Fig. 1H). Given that Acsl4 showed the highest upregulation in LGG1 and the highest downregulation in GBM7, we selected patient-derived glioma cells PL1 and PG7 for subsequent experiments, which were respectively isolated from discarded LGG1 and GBM7 specimens (Fig. 1E, Supplementary Fig. 1B). Additionally, we also detected the protein levels of Acsl4 among various glioma cell lines such as U87, U251, T98, PL1, and PG7, in contrast with normal human astrocytes (NHAs) in culture (Supplementary Fig. 1C). Our general assumption was that GBM might escape ferroptosis via genetic deficiency of Acsl4, which is essential for glioma lipidomics and serves as a vital ferroptosis marker in glioma.

### Acsl4 participated in mitochondrial-morphology regulation in ferroptosis

**Fig. 2:**
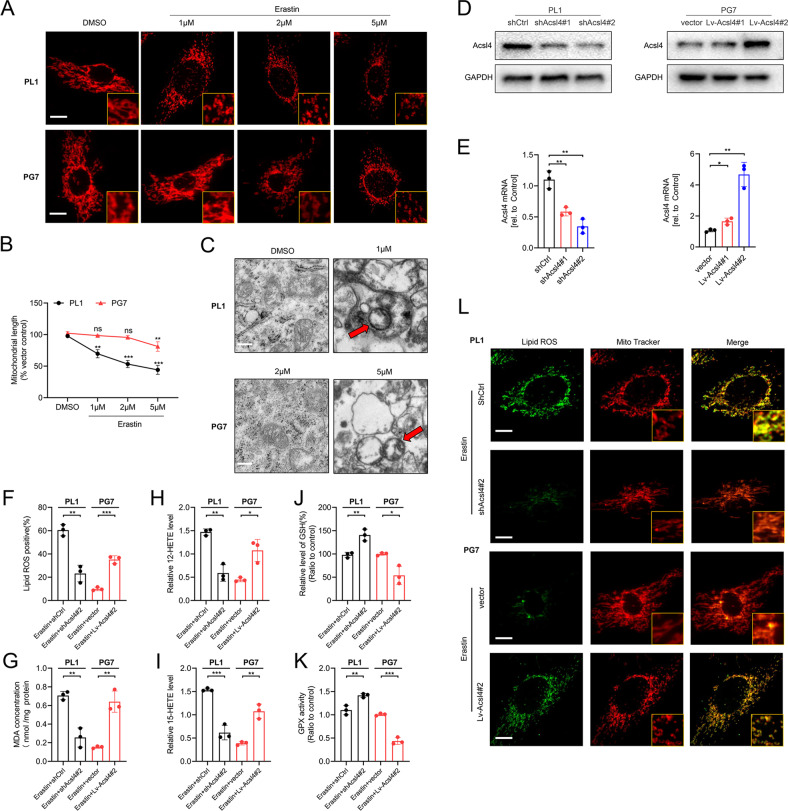
Acsl4 participates in mitochondrial-morphology regulation in ferroptosis. **A** Representative images by immunofluorescence show mitochondrial morphology in PL1 and PG7 cells treated with erastin in a dose-dependent manner (6 h). Scale bar: 10 μm. **B** The mean length of mitochondria in PL1 and PG7 cells treated with erastin dose dependently (6 h). Data indicated as mean ± S.D. (*n* = 3 experiments). **C** Representative transmission electron microscopy images show morphology of mitochondria in PL1 and PG7 cells under erastin treatment (6 h). Mitochondria showed the increased membrane density and shrunken morphology (red arrows). Scale bar: 2 μm. **D** Acsl4 protein expression levels in Acsl4 shRNA-mediated knockdown PL1 cells and Acsl4-overexpression PG7 cells were determined by western blot. **E** Acsl4 mRNA expression levels in Acsl4 shRNA-mediated knockdown PL1 cells and Acsl4-overexpression PG7 cells were determined by qPCR. Data indicated as mean ± S.D. (*n* = 3 experiments). **F**–**K** Intracellular ROS, MDA, 12-HETE, 15-HETE, GSH, and GPX activity in PL1 cells after 1 μM erastin treatment and in PG7 cells after 2 μM erastin treatment (6 h). Data indicated as mean ± S.D. (*n* = 3 experiments). **L** Confocal images showed colocalization of oxidized lipids (green) and mitochondria (red). PL1 and PG7 cells were treated as indicated before and then stained with BODIPY C11 and MitoTracker. Scale bar: 10 μm. *p < 0.05, **p < 0.01, ***p < 0.001.

Given the mitochondrion is a main organelle for cellular oxidative phosphorylation and ROS production [19], we wished to unambiguously determine whether mitochondria were affected in Acsl4-dependent ferroptosis in glioma cells. We observed that compared with GBM cells, LGG cells were liable to show more mitochondrial fragmentation accumulation around the nucleus in a dose-dependent manner in response to erastin toxicity (Fig. 2A). Quantification of mitochondrial length changed significantly at 1 μM in PL1 cells but showed no difference until 5 μM in PG7 cells (Fig. 2B). Furthermore, transmission electronic microscopy (TEM) revealed that PL1 cells treated with 1 μM erastin and PG7 cells treated with 5 μM erastin for 6 h had shrunken mitochondria, collapsed outer membranes and less microvilli than cells treated with the previous dose (Fig. 2C). Therefore, we speculated that low Acsl4 expression caused GBM cells to maintain a network of tubules, a shape typical of healthy and functional mitochondria.

To investigate this possibility, we depleted PL1 cells Acsl4 of via short-hairpin ribonucleic acid (shRNA) knockdown, creating shAcsl4 cells, and stably transfected these into PG7 cells, creating Lv-Acsl4 cells. Protein and genetic levels of Acsl4 were detected via WB and quantitative reverse-transcription polymerase chain reaction (qRT-PCR; Fig. 2D, E). Since accumulation of lipid ROS is an end product of lipid peroxidation and a hallmark of ferroptosis in glioma, we estimated levels of lipid peroxidation using C11 BODIPY 581/591. In PL1 cells, erastin reduced ROS accumulation by roughly two-third, whereas in PG7 cells, it had tripled (Fig. 2F). We verified with MDA, an independent probe (Fig. 2G). Moreover, both 12-HETE and 15-HETE levels were reduced when Acsl4 was knocked down in PL1 cells and increased when Acsl4 was overexpressed in PG7 cells (Fig. 2H, I). Additionally, as important antioxidants, reduced form GSH and GPX activity also confirmed the results (Fig. 2J, K). Furthermore, consistent with a prior study confirming subcellular localization of a lipid ROS probe via confocal-fluorescence microscopy (CFM), we found that in both PL1 and PG7 cells treated with erastin, the oxidized probe appeared in a distribution significantly colocalized with mitochondria and with the plasma membrane, with relatively high expression of Acsl4 (Fig. 2L). More importantly, the mitochondrial network of PL1–shAcsl4 cells was more elongated than that of PL1–shctrl cells, while PG7–Lv-Acsl4 cells became more fragmented and less elongated than PG7–vector cells.

Therefore, we concluded that the mitochondrion was a primary site of Acsl4-dependent ferroptosis in glioma cells and that mitochondrial morphology could be affected by expression of Acsl4 in glioma ferroptosis.

### Drp1 phosphorylation was essential for Acsl4-dependent ferroptosis

**Fig. 3:**
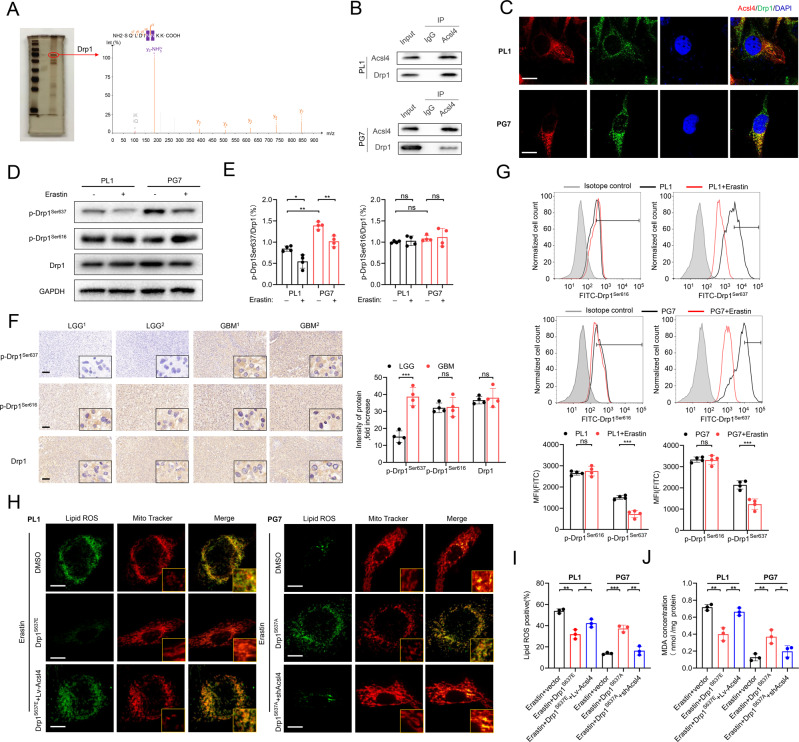
Drp1 phosphorylation is essential for Acsl4-dependent ferroptosis. **A** SDS-PAGE silver staining showed typical pull-down results of Acsl4 after incubation with PL1 cell lysate. Mass spectrometry identified the band framed in the oval as Drp1. **B** The interaction between Acsl4 and Drp1 was confirmed by co-immunoprecipitation in PL1 and PG7 cells. **C** Confocal images showed colocalization of Acsl4 (red) and Drp1 (green) in PL1 and PG7 cells. Nuclei were counterstained with Hoechst (blue). Scale bars: 10 μm. **D**, **E** Expression levels of p-Drp1^Ser637^, p-Drp1^Ser616^, and Drp1 were determined by western blot in PL1 and PG7 cells in the presence or absence of erastin (5 μM, 6 h). Drp1 was used as a loading control of two types p-Drp1. GAPDH was used as control. Data indicated as mean ± S.D. (*n* = 4 experiments). **F** Representative images of IHC staining of p-Drp1^Ser637^, p-Drp1^Ser616^, and Drp1 in two pairs LGG and GBM tissues. Scale bars: 100 μm. Data indicated as mean ± S.D. (*n* = 4 experiments). **G** Flow cytometric analysis of p-Drp1^Ser637^ and p-Drp1^Ser616^ levels in PL1 and PG7 cells. Isotype control was set in gray. The histogram shows mean fluorescence intensity (MFI) values for control and erastin-treated cells. Data indicated as mean ± S.D. (*n* = 4 experiments). **H** Confocal images showed colocalization of oxidized lipids (green) and mitochondria (red). PL1 cells of indicated groups were treated with erastin (1 μM, 6 h), and PG7 cells of the indicated groups were treated with erastin (2 μM, 6 h); then cells were stained with BODIPY C11 and MitoTracker. Scale bar: 10 μm. **I**–**J** Intracellular ROS and MDA level in PL1 and PG7 cells treated as indicated before. Data indicated as mean ± S.D. (*n* = 3 experiments). *p < 0.05, **p < 0.01, ***p < 0.001.

To establish potential regulators of Acsl4, we identified the proteins pulled down (Supplementary Table 2). Compared with control, we distinctly observed enrichment of proteins and a prominent band resolved at approximately 83 kDa, as shown in the sodium dodecyl sulfate-polyacrylamide gel electrophoresis (SDS-PAGE) images in Fig. 3A. Using mass spectrometry (MS), we examined whole-eluted samples without bias. Next, we detected Drp1 as the most enriched protein. As expected, Drp1 was immunoprecipitated by sepharose-A coated with Acsl4 antibodies but not by immunoglobulin G (IgG; Fig. 3B), which was also confirmed by CFM (Fig. 3C).

Drp1, which is known for its role in regulating mitochondrial morphology [20], is related to erastin-induced ferroptosis in melanoma cells [21]. Therefore, we detected expression patterns of Drp1 in LGG and GBM samples using WB. Interestingly, we found no difference in Drp1 expression between LGGs and GBMs (Supplementary Fig. 2A, B). As is well known, Drp1 activity in glioma cells is regulated by post-translational modifications, mainly by phosphorylation: while serine 616 (Ser616) residue is an activation site, Ser637 residue is a repression site [22, 23]. We next determined phosphorylated Drp1 (p-Drp1) levels at Ser616 and Ser637 in primary LGG and GBM clinical specimens. In every model we tested, p-Drp1 (Ser637) levels were significantly increased in GBMs compared with LGGs, while p-Drp1 (Ser616) and Drp1 levels did not differ between the two specimen types, suggesting that GBM cells had attenuated activity of Drp1. Moreover, erastin reduced Drp1 Ser637 expression in LGG and GBM specimens (Fig. 3D–G), indicating that inactivation of Drp1 by phosphorylation at Ser637 was suppressed in erastin-induced ferroptosis. Then, to determine whether Drp1 phosphorylation was relevant to Acsl4-dependent ferroptosis, we generated a gain-of-function Drp1 containing both S637E (to mimic inhibitory phosphorylation) mutations in PL1 cells and S637A (to block inhibitory phosphorylation–dephosphorylation) mutations in PG7 cells (Supplementary Fig. 2C, D). Notably, in western blot assay, Acsl4 expression was found to be positively correlated with Drp1 activity following erastin treatment. CFM revealed that the morphology of mitochondria in PL1–Drp1^S637E^ cells became more filamentous. The alterations in lipid mediators and mitochondrial morphology were reversed when Acsl4 was overexpressed in PL1–Drp1^S637E^ cells (Fig. 3H–J, Supplementary Fig. 3A–D), indicating that phosphorylation of Drp1 at Ser637 in LGG cells suppressed Acsl4-dependent ferroptosis. Correspondingly, PG7–Drp1^S637A^ cells tended to show a fragmented mitochondrial phenotype. However, when we knocked down Acsl4 in PG7–Drp1^S637A^ cells, those surrogate markers were once again reversed (Fig. 3H–J, Supplementary Fig. 3A–D), suggesting that dephosphorylation of Drp1 at Ser637 in GBM cells strongly induced Acsl4-dependent ferroptosis. Of note, one previous study have suggested that STING1 promotes ferroptosis through MFN1/2-dependent mitochondrial fusion in pancreatic cancer cells [24]. We then tested the effect of MFN1/2 on Acsl4, and found that the downregulation of MFN1/2 level did not change the expression of Acsl4 in the context of gliomas (Supplementary Fig. 4A- D).

Overall, Drp1 dephosphorylating at Ser637 reduced mitochondrial filamentation, which was essential for Acsl4-dependent ferroptosis in glioma cells.

### Hsp90 regulated Drp1 phosphorylation via calcineurin in gliomas

**Fig. 4:**
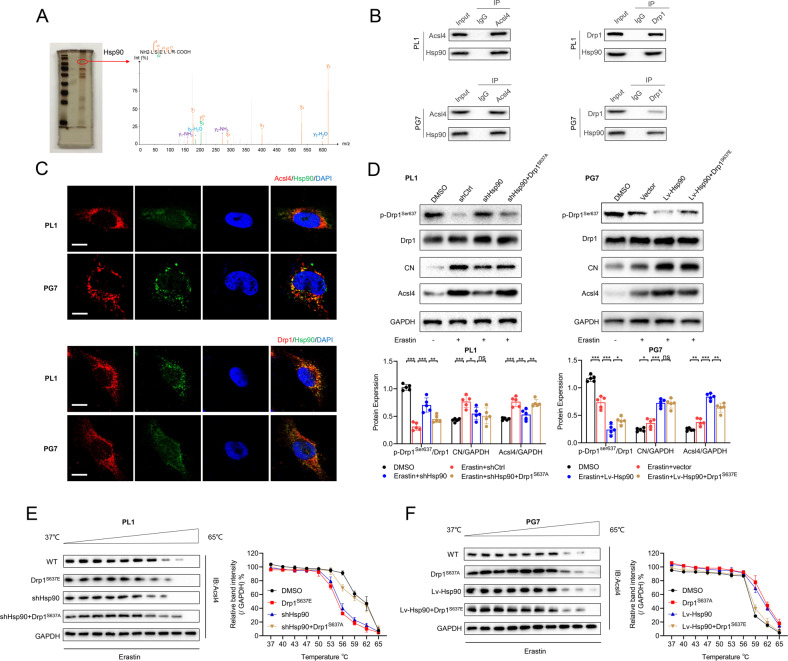
Hsp90 regulates Drp1 phosphorylation via calcineurin in gliomas. **A** SDS-PAGE silver staining showed typical pull-down results of Acsl4 after incubation with PL1 cell lysate. Mass spectrometry identified the band framed in the oval as Hsp90. **B** The interaction between Acsl4, Drp1, and Hsp90 was confirmed by co-immunoprecipitation in PL1 and PG7 cells. **C** Confocal images showed colocalization of Acsl4 (red) and Hsp90 (green), Drp1 (red), and Hsp90 (green) in PL1 and PG7 cells. Nuclei were counterstained with Hoechst (blue). Scale bars: 10 μm. **D** Expression levels of proteins in Hsp90-Acsl4 pathway were determined by western blot in the indicated groups. Data indicated as mean ± S.D. (*n* = 5 experiments). **E**, **F** Thermal stabilization of Acsl4 in PL1 and PG7 cells of indicated groups was determined following standard cellular thermal shift protocol with heat treatment from 37 °C to 65 °C. (PL1 cells: erastin 1 μm, PG7 cells: erastin 2 μm). Data indicated as mean ± S.D. (*n* = 3 experiments). *p < 0.05, **p < 0.01, ***p < 0.001.

Through MS, we found that Hsp90 as well as Drp1 interacted with Acsl4 (Fig. 4A). Hsp90, as a global regulator of tumor cell metabolism in mitochondria including oxidative phosphorylation and redox networks, is defined as a common regulatory node in both necroptosis and ferroptosis [25, 26]. Co-immunoprecipitation (Co-IP) experiments in PL1 and PG7 cells showed that Acsl4, Drp1, and Hsp90 interacted with each other (Fig. 4B), and confocal images showed that Hsp90 colocalized with Acsl4 and Drp1 in the mitochondrial outer membrane (Fig. 4C).

In many types of cells, calcineurin (CN) dephosphorylates Drp1 at Ser637 [27]; and Hsp90 binds to CN and stimulates its activity [28]. Therefore, we verified Hsp90’s effects on CN and Drp1 in glioma, notably on the Drp1–Acsl4 axis in erastin-induced ferroptosis. In PL1 cells, Hsp90 knockdown increased Drp1 (Ser637) phosphorylation while decreasing CN and Acsl4 levels. Of note, Drp1 expression remained constant. Similarly, Hsp90 overexpressing in PG7 cells triggered the Drp1–Acsl4 axis (Fig. 4D). Subsequently, we continued to explore possible mechanisms by which Hsp90- Drp1 upregulated Acsl4 in vitro. First, when we used actinomycin D, a transcription inhibitor to treat cells, we found that neither phosphorylation of Drp1 nor the changes of Hsp90 expression had any significant effect on the mRNA levels of Acsl4 (Supplementary Fig. 3E, F). This suggested that regulation of Acsl4 by Hsp90-Drp1 is not based on the mRNA levels and instead indicated that the stability of Acsl4 protein might be regulated by Hsp90-Drp1. As Hsp90 functions by promoting the structural stability of its client proteins [29], we employed cellular thermal shift assay to provide explicit evidence for our assumption. The results showed that, compared with DMSO groups, Drp1^S637E^ and downregulation of Hsp90 significantly decreased the thermal stabilization, while the Drp1^S637A^ and overexpression of Hsp90 significantly increased the thermal stabilization (Fig. 4E, F), suggesting a priorly higher binding of Hsp90- Drp1 with Acsl4 protein.

### Promotion of the Hsp90–Acsl4 pathway enhanced erastin sensitivity in vitro

**Fig. 5:**
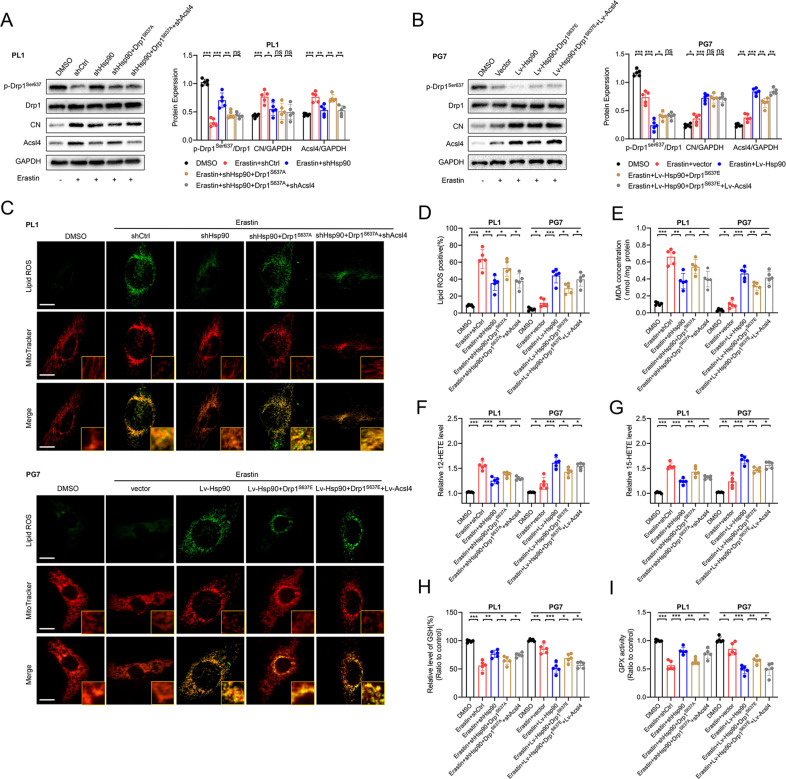
Promotion of the Hsp90-Acsl4 pathway enhances Acsl4-dependent ferroptosis. **A**, **B** Expression levels of proteins in Hsp90-Acsl4 pathway were determined by western blot in the indicated groups. Data indicated as mean ± S.D. (*n* = 5 experiments). **C** Confocal images showed colocalization of oxidized lipids (green) and mitochondria (red). PL1 cells of indicated groups were treated with erastin (1 μM, 6 h), and PG7 cells of the indicated groups were treated with erastin (2 μM, 6 h); then the cells were stained with BODIPY C11 and MitoTracker. Scale bar: 10 μm**. D**–**I** Intracellular ROS, MDA, 12-HETE,15-HETE levels, and GSH and GPX activity in PL1 and PG7 cells treated as indicated before. Data indicated as mean ± S.D. (*n* = 5 experiments). *p < 0.05, **p < 0.01, ***p < 0.001.

We sought to determine the relevance of the Hsp90–Acsl4 pathway in erastin-induced ferroptosis in vitro. First, we investigated the effect of Hsp90 on Drp1 and Acsl4 protein levels in erastin-induced ferroptosis. As shown in Fig. 5A, B, shHsp90 significantly promoted Drp1^Ser637^ level and inhibited CN and Acsl4 expression; meanwhile, Drp1 expression remained unchanged in PL1 cells. Similar results could be verified in PG7 cells.

Next, we examined whether Hsp90 could sensitize glioma cells to Acsl4-dependent ferroptosis. Figure 5D–G shows that Hsp90 reduced lipid ROS, MDA production, and 12- and 15-HETE levels in PL1 and PG7 cells. Hsp90 stimulation depleted GSH, while GPX activity was downregulated in glioma cells (Fig. 5H, I). Furthermore, CFM revealed that mitochondria extended throughout the cell body to sites distal from the nucleus when the Hsp90–Acsl4 pathway was downregulated, and they showed more fragmentation accumulation around the nucleus when this pathway was upregulated (Fig. 5C).

**Fig. 6:**
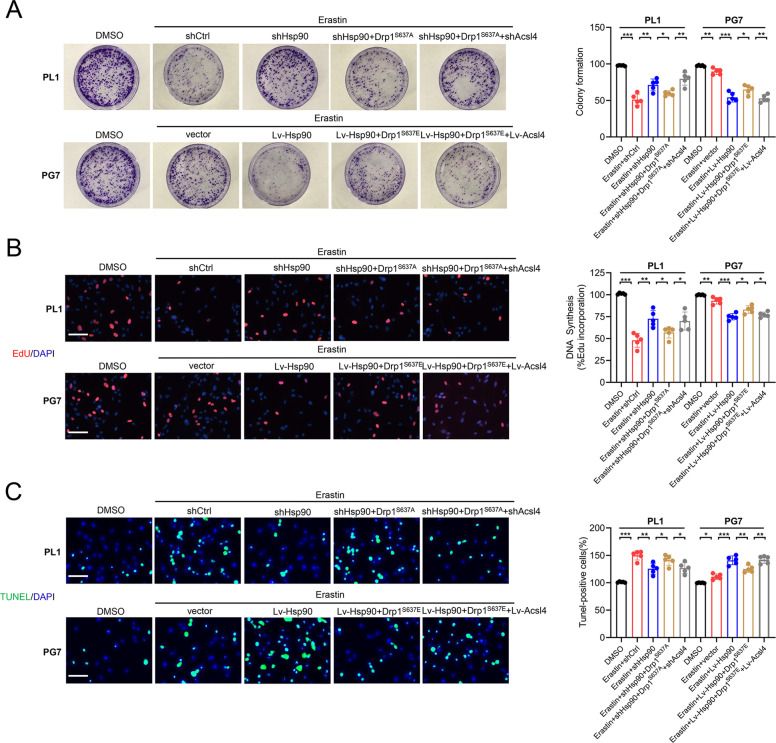
Promotion of the Hsp90-Acsl4 pathway enhances erastin sensitivity in vitro. **A** Colony formation assays in the indicated groups in PL1 cells after 1 μM erastin treatment and in PG7 cells after 2 μM erastin treatment (6 h). Data indicated as mean ± S.D. (*n* = 5 experiments). **B** EdU assays. Scale bar: 50 μm. Data indicated as mean ± S.D. (*n* = 5 experiments). **C** TUNEL assays. Scale bar: 50 μm. Data indicated as mean ± S.D. (*n* = 5 experiments). *p < 0.05, **p < 0.01, ***p < 0.001.

We further investigated the Hsp90–Acsl4 pathway mechanisms by studying the cytotoxic efficacy of erastin in glioma cells. Colony formation, EdU, and TUNEL experiments were performed to evaluate cell proliferation. These results showed that in all highly Acsl4-expressing cells (Lv-Hsp90, Drp1^S637A^, and Lv-Acsl4), the ability of erastin to inhibit cell proliferation was notably enhanced; meanwhile, in cells with reduced Acsl4 expression levels (shHsp90, Drp1^S637E^, and shAcsl4), this ability was comparably reduced (Fig. 6A–C). We also performed CCK-8 and cytotoxicity (LDH) assays to confirm the effect (Supplementary Fig. 5A–C). Of note, as stated before, the overexpression of Hsp90 and Drp1 dephosphorylation at Ser637 increased erastin-induced lipid peroxidation in glioma cells, but as indicated, this effect was completely reversed by ferroptosis inhibitors (ferrostatin-1), but not by inhibitors of apoptosis (ZVAD- FMK) (Supplementary Fig. 5D–I). Consequently, apoptosis could not be affected in PL1 and PG7 cells with changed Acsl4 levels. It was also proved by the increase of MMP, and the comparable expression levels of cleaved Caspase-3 and BCL2 family (Supplementary Fig. 6A–E). Therefore, these results confirmed that Hsp90–Acsl4 pathway upregulation promoted ferroptosis, thereby decreasing proliferation of glioma cells.

### Promotion of the Hsp90–Acsl4 pathway enhanced erastin sensitivity in vivo

To examine whether promotion of the Hsp90–Acsl4 pathway also increased tumor sensitivity to erastin in vivo, we first established mouse subcutaneous and orthotopic models via PG7 cells to confirm the sensitivity of parental GBM cells to different concentrations of erastin (Supplementary Fig. 7A). The anticancer impact of erastin was not noticeable until 15 mg (Supplementary Fig. 7B–F).

**Fig. 7:**
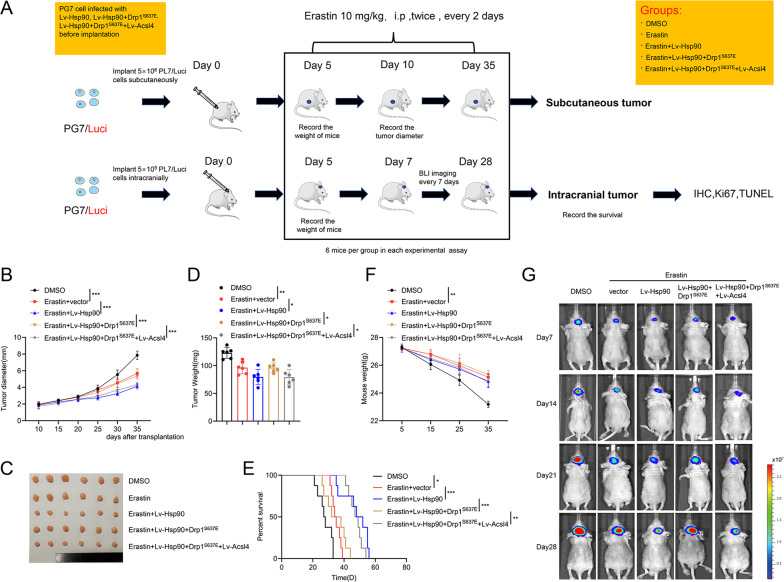
Promotion of the Hsp90-Acsl4 pathway enhances erastin sensitivity in vivo. **A** Mice were subcutaneously and intracranially xenografted with PG7 cells of different groups (5 × 10^6^/5 × 10^5^ cells) and treated intraperitoneally with erastin (10 mg kg^−1^ day^−1^ per mouse) or DMSO (0.3%) twice, every two days. **B** Diameter of subcutaneous tumors. Data indicated as mean ± S.D. (*n* = 6 mice per group). **C** Image of subcutaneous tumors. **D** Tumor weight of subcutaneous tumors. Data are indicated as mean ± S.D. (*n* = 6 mice per group). **E** Kaplan–Meier survival of mice. (*n* = 6 mice per group). **F** Weight of mice during the experiment. Data indicated as mean ± S.D. (*n* = 6 mice per group). **G** Bioluminescence imaging was performed on days 7, 14, 21, and 28 after implantation. *p < 0.05, **p < 0.01, ***p < 0.001.

Next, we assessed the therapeutic value of Acsl4 overexpression on GBM cells in vivo. Five days after PG7 implantation, mice were treated i.p. with erastin (10 mg/kg^−1^/day^−1^ per mouse) or DMSO (0.3%) every 2 days (Fig. 7A). Tumor inhibition was greater when erastin was paired with Hsp90 or Acsl4 overexpression. Drp1^S637E^ inhibited the growth of erastin-treated Lv-Hsp90 PG7 cells to the same degree that it did to erastin-treated PG7 tumors (Fig. 7B–D). Mice receiving combined treatment showed considerably smaller tumor volume than other mice (Fig. 7G) and had dramatically prolonged lifespans (Fig. 7E). Orthotopic glioblastoma development markedly decreased mouse weight, which was mitigated by erastin administration (Fig. 7F).

**Fig. 8:**
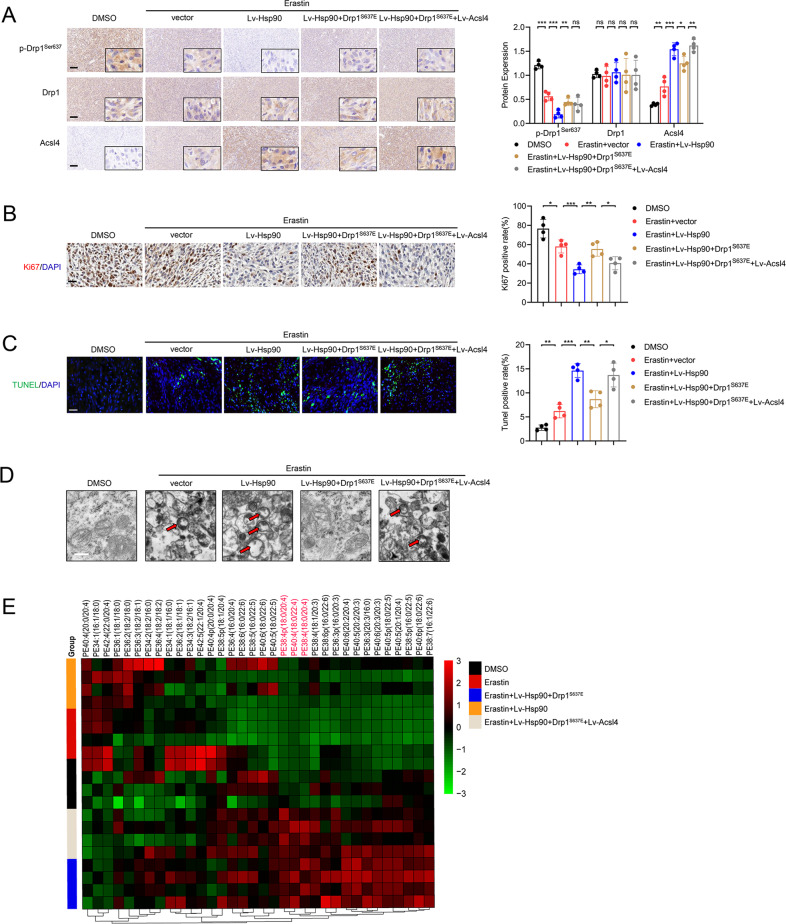
Promotion of the Hsp90-Acsl4 pathway enhances erastin sensitivity in vivo. **A** IHC assay for p-Drp1^Ser637^, Drp1, and Acsl4. Scale bar: 100 μm. Data indicated as mean ± S.D. (*n* = 4 mice per group). **B** IHC assay for Ki67. Scale bar: 50 μm. Data indicated as mean ± S.D. (*n* = 4 mice per group). **C** TUNEL assay. Scale bar: 50 μm. Data indicated as mean ± S.D. (*n* = 4 mice per group). *p < 0.05, **p < 0.01, ***p < 0.001. **D** Representative transmission electron microscopy images revealed mitochondrial morphology of different groups. Scale bar: 1 μm. **E** Heat map of all major PE species with hierarchical clustering of the groups DMSO, Erastin, Erastin+ Lv-Hsp90, Erastin + Lv-Hsp90 + Drp1^S637E^, Erastin+ Lv-Hsp90 + Drp1^S637E^ + Lv-Acsl4.

We next assessed levels of primary Hsp90–Acsl4 pathway proteins in mouse tumors using IHC. Consistent with the in vitro results, Hsp90 overexpression mitigated p-Drp1^Ser637^ levels and enhanced Acsl4 expression, whereas Drp1 level did not change significantly (Fig. 8A). Additionally, mice with Hsp90–Acsl4 overexpression showed decreased Ki-67 levels (Fig. 8B). TUNEL assays demonstrated that erastin mildly promoted cell death in vivo. Hsp90 and Acsl4 overexpression intensified erastin-induced PG7 cell death, but Drp1^S637E^ limited this effect (Fig. 8C). In addition, typical mitochondrial changes and lipidomic changes caused by ferroptosis were found in the erastin, erastin + Lv-Hsp90, and erastin + Lv-Hsp90 + Drp1^S637E^ + Lv-Acsl4 groups. These changes were alleviated in the erastin + Lv-Hsp90 + Drp1^S637E^ group (Fig. 8D, E). Overall, our findings suggest Acsl4 as a possible therapeutic target to boost erastin’s advantages.

## Discussion

Herein, we described an underlying mechanism for the regulation of ferroptosis between different grades of gliomas: LGGs could have a considerable effect on the decision-making because ferroptosis was more likely to happen in LGGs due to the high Acsl4 expression levels of LGGs. Studies show that curing glioma with existing treatments is difficult, but it is obvious that with appropriate management, patients with LGG have better prognosis and survival than those with GBM [1]. Previous research has linked ferroptosis suppression to glioma development and poor prognosis [30–32]. Consistent with the prior studies, we observed reduced viability and inhibited proliferation of glioma cells in erastin-induced ferroptosis. Considering that the complex central nervous system is under frequent oxidative stress [33], this mechanism might represent the role of ferroptosis in the prognosis and treatment of gliomas.

Stockwell et al. elucidated that ferroptosis is a form of RCD that occurs as a consequence of lethal-lipid peroxidation [34]. Furthermore, Xie Y noted that changes in mitochondria morphology, as representative RCD-executing organelles, distinguish ferroptosis from other forms of programmed death [4]. In this study, LGGs were more sensitive to ferroptosis based on lipidomics and mitochondrial morphology. Database analyses demonstrated that Acsl4 was genetically expressed at relatively high levels in LGGs *versus* GBMs, as validated by protein levels. Kagan has revealed that Acsl4 is dedicated to shaping the lipidomic of cells by playing an important role in determining sensitivity *versus* resistance to ferroptosis [16]. Therefore, we hypothesized that the reduction of ferroptosis in GBMs might be attributable to a reduction in Acsl4 expression. Transfection of shAcsl4 or Acsl4 overexpression plasmids into two primary glioma cell lines, PL1 and PG7, confirmed the role of Acsl4 in glioma ferroptosis. Lipid ROS, MDA, 12-HETE, 15-HETE, and GSH and GPX activities, which are main products of cellular oxidative metabolism, are regarded as Acsl4-dependent ferroptosis indicators. The results demonstrated that Acsl4 could be the component that differentiates ferroptotic sensitivity between different grades of gliomas.

Mitochondrial morphology is tightly coordinated in association with the cell death, and this has been studied extensively in various tumors [35–37]. As in other RCDs such as autophagy and apoptosis, the role of mitochondrial morphology in ferroptosis is discussed heatedly. Neitemeier et al. observed time-dependent changes in mitochondrial morphology in response to erastin toxicity, which comprised mitochondrial fragmentation and accumulation around the nucleus [5]. Du et al. suggested that suppression of FXN expression significantly enhanced erastin-induced cell death resulted in dramatic mitochondria morphological damage including enhanced fragmentation and vanished cristae [38]. However, Li et al. found that STING1 promotes ferroptosis through MFN1/2-dependent mitochondrial fusion [24]. Opposite results on the roles of mitochondria in ferroptosis may be due to the different cell lines and different concentration of erastin. The molecular mechanisms by which oncogenic signaling pathways could alter mitochondrial dynamics in glioma ferroptosis deserve further investigation.

Hsp90 mainly protect cells against harmful stimuli by stabilizing unfolded or misfolded peptides and by repairing or promoting the degradation of denatured proteins [25, 39]. The role of the Hsp90 in cell death is disputable. In previously published studies [26, 40], Hsp90 was found to promote the ferroptosis process by accelerating the degradation of GPX4 in the chaperone-mediated autophagy(CMA) pathway. However, targeting Hsp90 has been shown to be an effective anticancer therapy in gliomas because inhibition of Hsp90 can suppress the proliferation and survival of glioma cells [41, 42]. Thus, the effects of Hsp90 might be context specific and stimulation dependent. In our study, we found that Hsp90-dependent Drp1 dephosphorylation promoted Acs4 expression by binding and stabilizing Acsl4, which could sensitize glioma cells to erastin-induced ferroptosis. Hsp90 therefore served as a positive rather than a negative regulator of ferroptosis due to molecular-pathway differences.

Of late, several clinical drugs are drawing increasing appreciation because of their capacity for inducing ferroptosis in cancer cells. Erastin is the prototype ferroptosis inducer that can reduce GSH levels by inhibiting system Xc- directly [43]. Sulfasalazine (SAS), an anti-inflammatory drug, can induce ferroptosis in a series of cancer cell lines (HT-1080, BJeLR, Calu-1, 143B) and has been used in combination therapy to enhance the therapeutic efficacy of other chemotherapeutics against glioma [44, 45]. Sorafenib, a clinically approved multi-kinase inhibitor for the treatment of advanced carcinoma, induces ferroptosis independently [44]. Other small-molecule inducers of ferroptosis include FIN56, which degrades GPX4, binds to squalene synthase (SQS), and depletes the antioxidant coenzyme Q_10_ (CoQ10) [46]; statins, which inhibit 3-hydroxy-3-methyl-glutaryl-CoA reductase (HMGCR), decrease GPX4 levels, and block biosynthesis of CoQ10[47]; and BAY 87–2243, which inhibits mitochondrial complex I (MC-I) [21]. Most importantly, erastin and its derivatives have successfully treated diffuse large B-cell lymphoma (DLBCL) in a SUDHL6 cell xenograft animal model [48].

Notably, the dose of erastin (10 mg/kg, i.p., twice every other day) we explored and used in treating glioma was lower than that in other tumors reported before [49–51]. The low-dose treatment not only had no adverse events, but also had remarkable anti-tumor effects, which adequately proved that the induction of ferroptosis is practical and feasible in viable anti-glioma tactics. Furthermore, promotion of Hsp90-Acsl4 contributed to efficient tumor inhibition and obvious survival improvement, due to the increasing sensitivity of glioma to erastin-induced ferroptosis.

In summary, erastin-induced ferroptosis indicates a prospective molecular approach to target gliomas. Our study uncovered an important role of Acsl4 in predicting patient prognosis due to its high inducibility in glioma ferroptosis. Furthermore, we suggested an underlying regulatory mechanism of Hsp90-Acsl4 as a potential therapeutic pathway to enhance the effect of ferroptosis-inducing therapy for gliomas.

## Supplementary information

Supplement

original western blots

## Data Availability

The data used to support the findings of this study are available from the corresponding author upon request.
