# Supplementary material for: Hsp90 induces Acsl4-dependent glioma ferroptosis via dephosphorylating Ser637 at Drp1
Source: Cell Death Dis. 2022 Jun 13;13(6):548. doi: 10.1038/s41419-022-04997-1 (PMC9192632; doi:10.1038/s41419-022-04997-1)
Supplement: Supplementary file 1 — Supplement [file 41419_2022_4997_MOESM1_ESM.docx]

**Supplementary Materials**


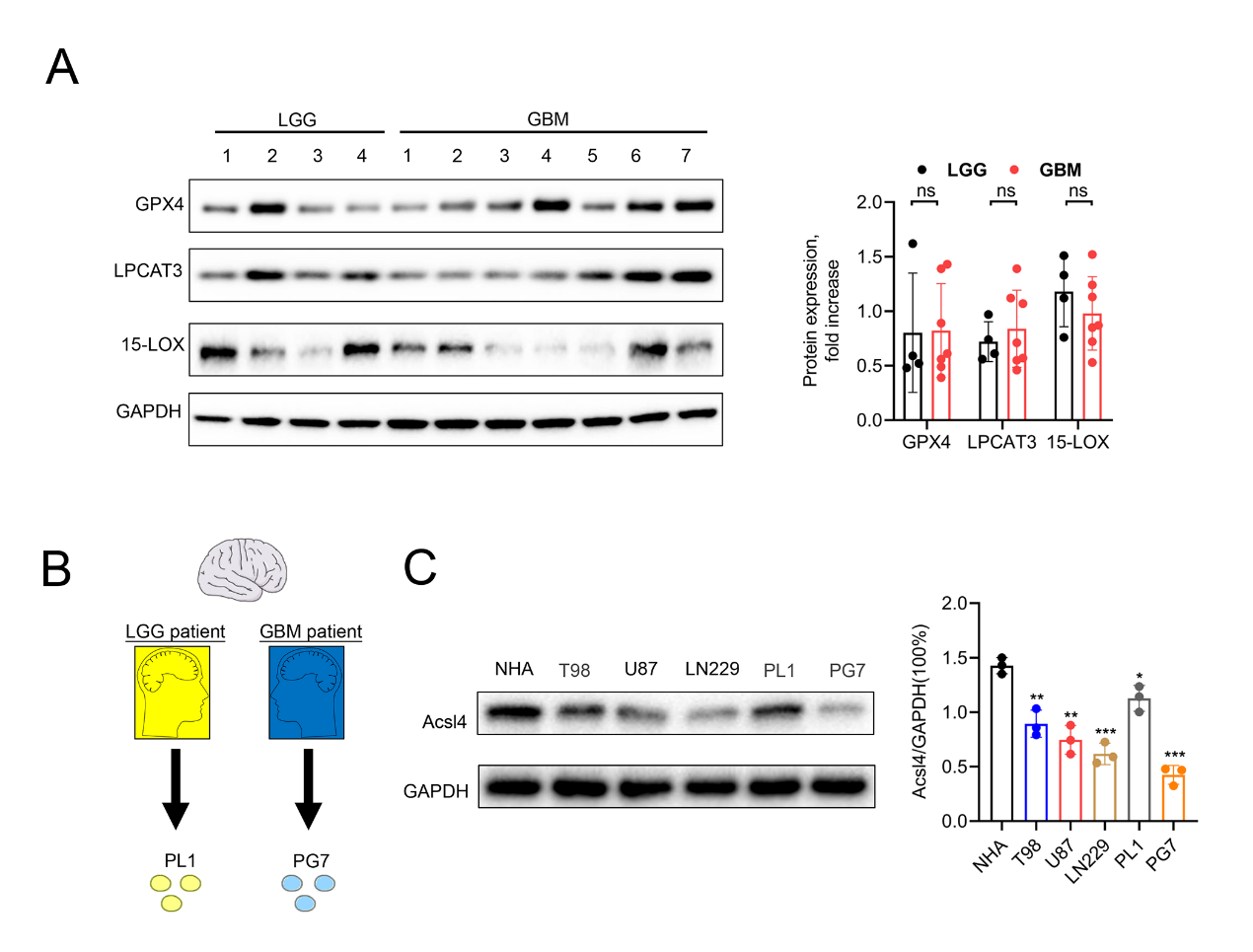


**Supplementary Fig.1 The process and reason of selecting Acsl4, PL1 and PG7 cells in this study.**

**(A)** GPX4, LPCAT3, and 15-LOX protein levels in in human glioma (LGG, n = 4; GBM, n = 7) samples. GAPDH was used as control in western blot assays. **(B)** Schematic process to acquire primary glioma cells-PL1 and PG7 cells. **(C)** Acsl4 protein expression levels in various human glioma cell lines. Data indicated as mean ± S.D. (n = 3 experiments). * p ≤ 0.05,**p ≤ 0.01,***p ≤ 0.001.


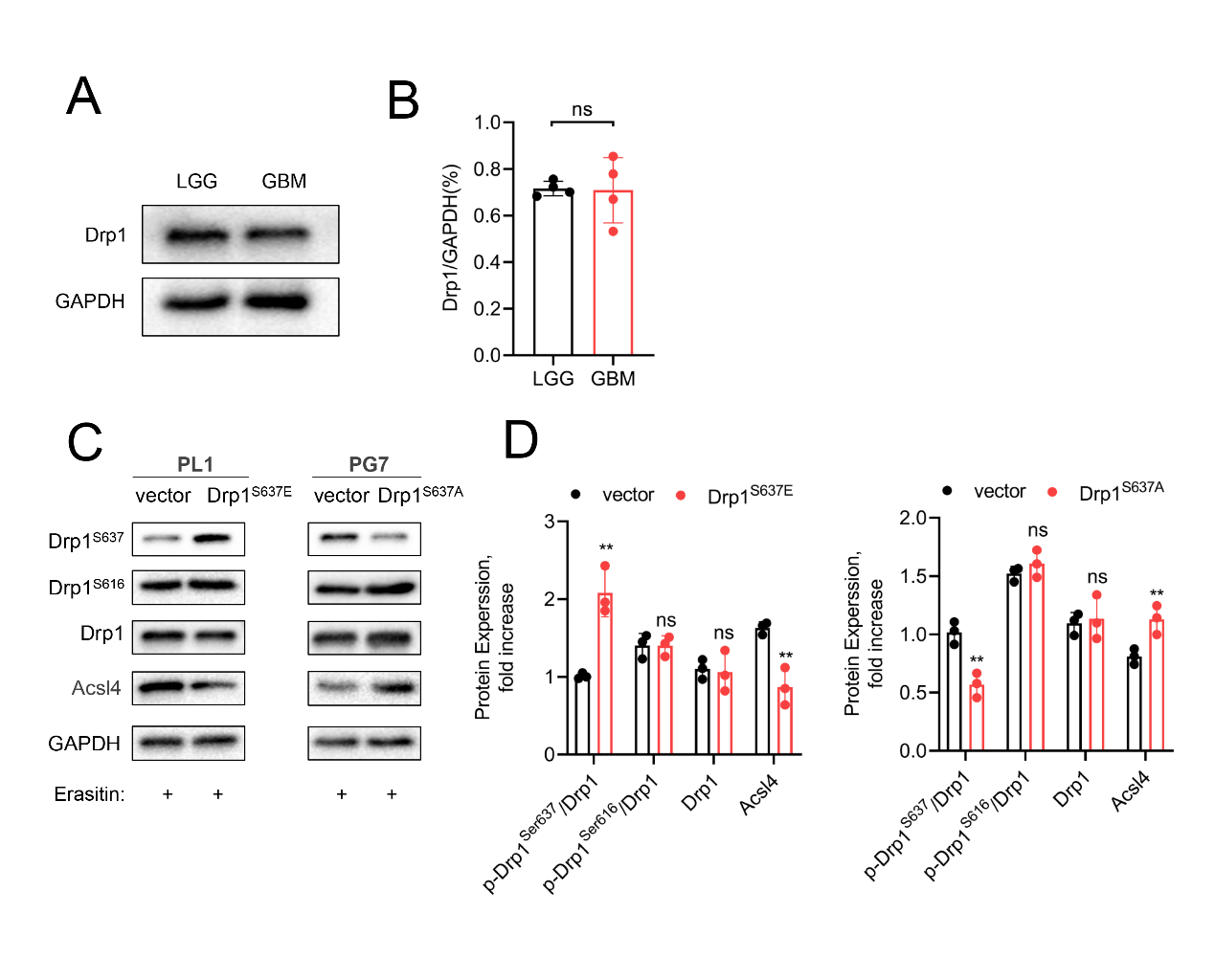


**Supplementary Fig.2 Expression of Drp1-Acsl4 axis protein in PL1-Drp1^S637E^ and PG7-Drp1^S637A^ cells.**

**(A-B)** Drp1 protein levels in LGG and GBM samples. Data indicated as mean ± S.D. (n = 4 experiments). **(C-D)** p-Drp1Ser637, p-Drp1Ser616, Drp1, and Acsl4 protein levels in PL1-Drp1S^637E^ and PG7-Drp1^S637A^  cells. GAPDH was used as control. Data indicated as mean ± S.D. (n = 3 experiments). *p < 0.05, **p < 0.01, ***p < 0.001.


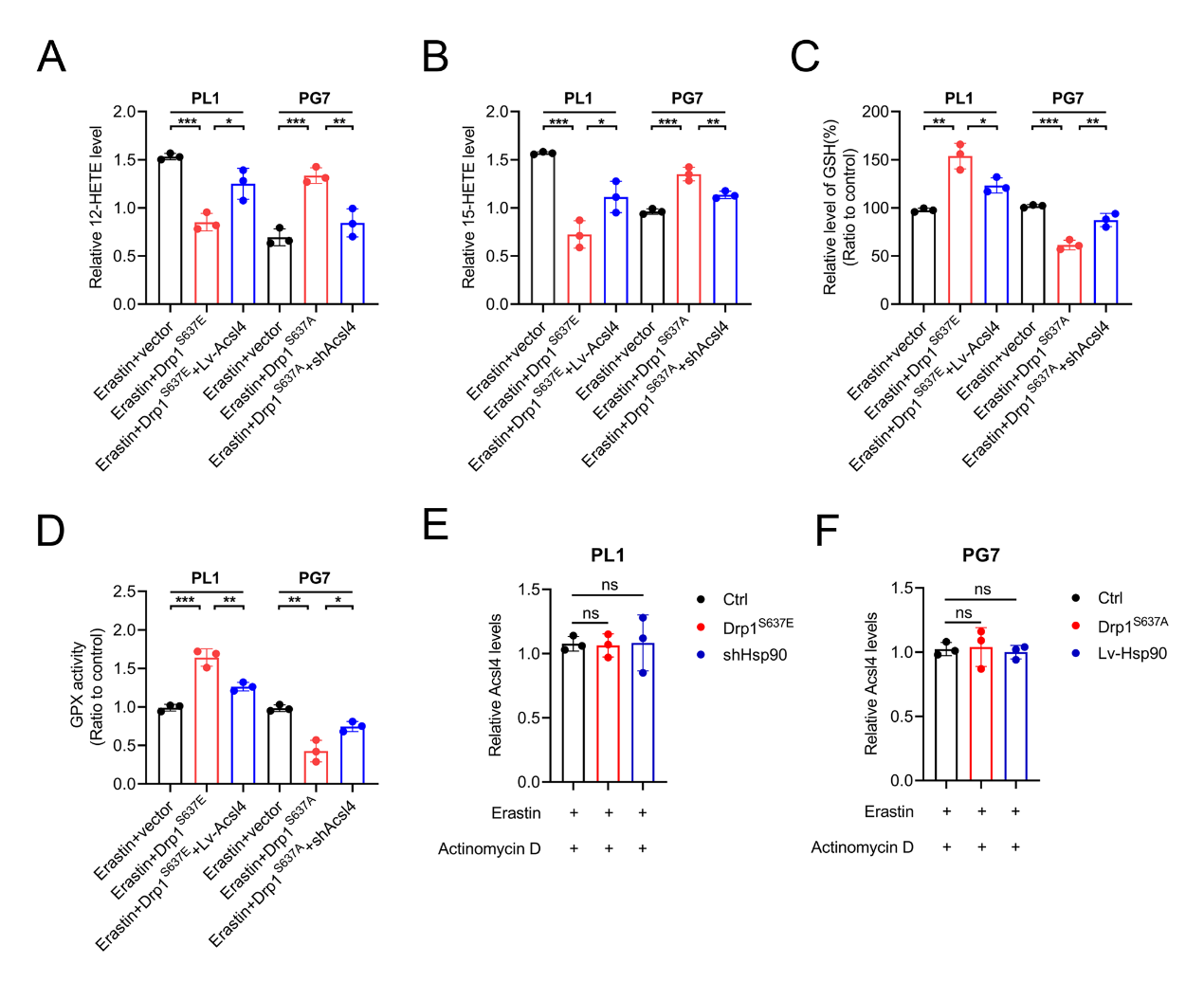


**Supplementary Fig.3 Drp1 phosphorylation are essential for Acsl4-dependent ferroptosis.**

**(A-D)** Intracellular 12-HETE, 15-HETE level, GSH, and GPX activity in PL1 cells of the indicated groups after 1 μM erastin treatment (6 h), and in PG7 cells of the indicated groups after 2 μM erastin treatment (6 h). Data indicated as mean ± S.D. (n = 3 experiments). **(E-F)** qRT-PCR analysis of Acsl4 mRNA expression in PL1 cells of the indicated groups treated with erastin (1 μm) and actinomycin D (5 μg/ml), and in PG7 cells of the indicated groups treated with erastin (2 μm) and actinomycin D (5 μg/ml). Data indicated as mean ± S.D. (n = 3 experiments). *p < 0.05, **p < 0.01, ***p < 0.001.


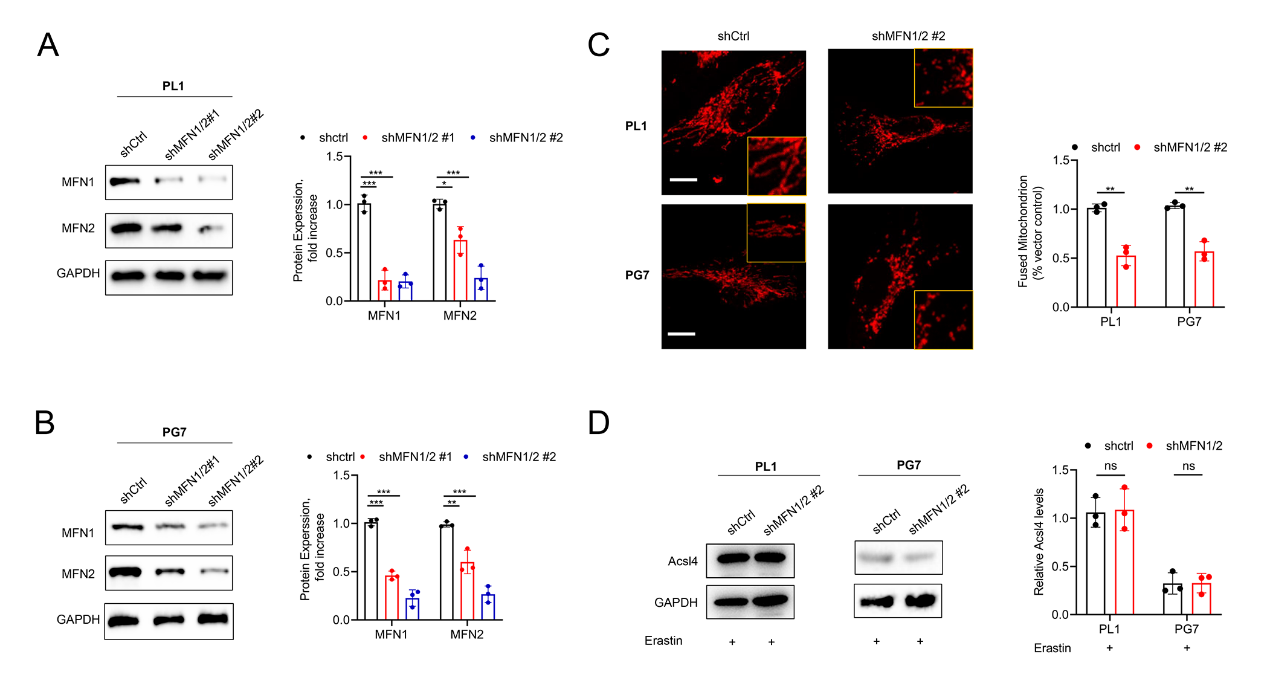
**Supplementary Fig.4 The downregulation of MFN1/2 level is not associated with the expression of Acsl4 in the context of gliomas.**

**(A-B)** MFN1/2 protein expression levels in MFN1/2 shRNA-mediated knockdown PL1 and PG7 cells were determined by western blot. (**C)** Representative images by immunofluorescence show mitochondrial morphology in PL1 and PG7 cells. **(D)** Expression levels of Acsl4 was determined by western blot in shCtrl and shMFN1/2. PL1 cells were treated with 1 μM erastin and PG7 cells were treated with 5 μM erastin. GAPDH was used as control in western blot assays. Data indicated as mean ± S.D. (n = 3 experiments). Scale bar: 10 μm. *p < 0.05, **p < 0.01, ***p < 0.001.


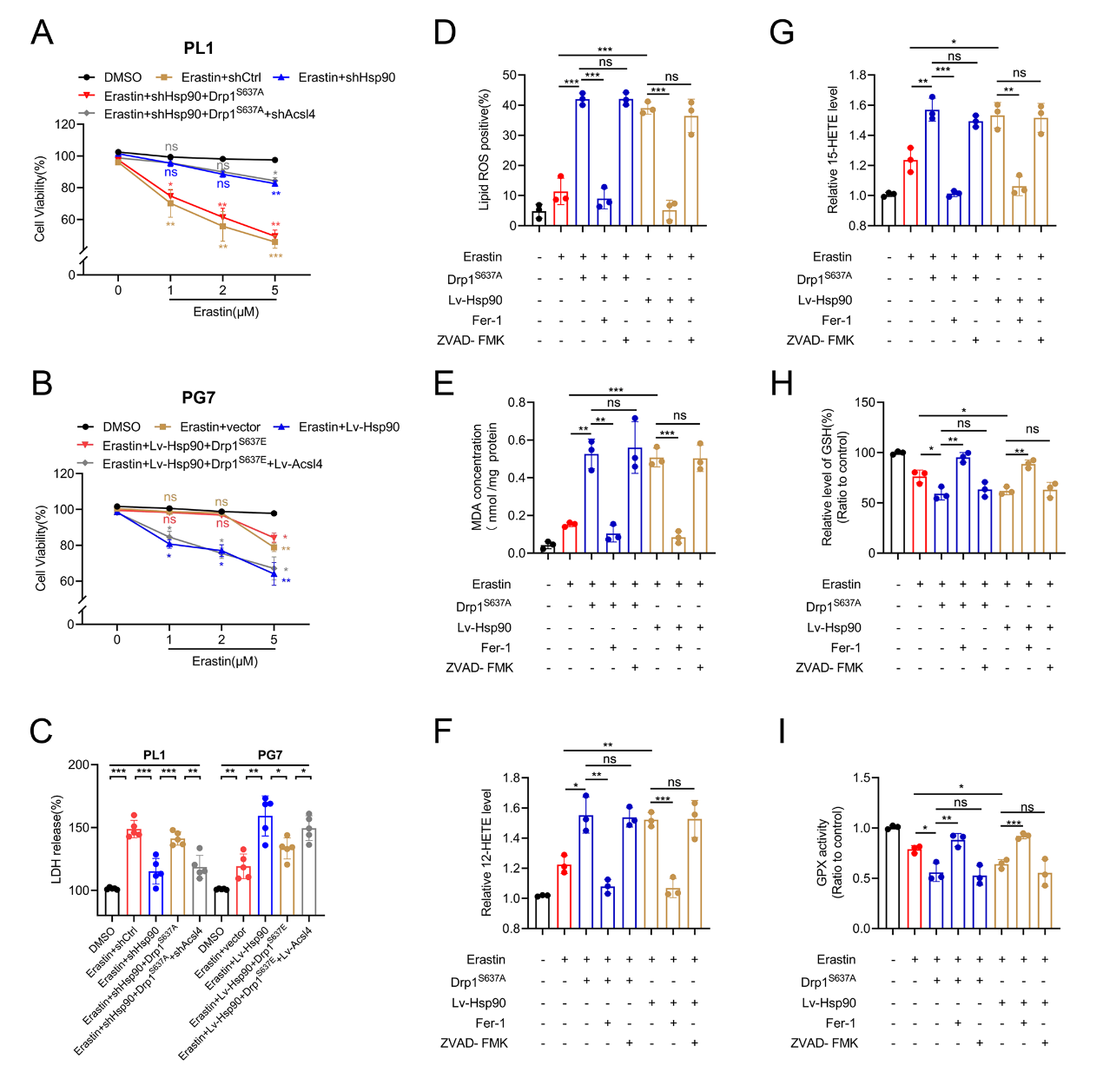


**Supplementary Fig.5 Promotion of the Hsp90-Acsl4 pathway enhances erastin sensitivity *in vitro*.**

**(A-B)** CCK-8 assay. PL1 cells were treated with 1 μM erastin and PG7 cells were treated with 2 μM erastin for 6 h. Data indicated as mean ± S.D. (n = 5 experiments). **(C)** LDH assay in the indicated groups as treated before. Data indicated as mean ± S.D. (n = 5 experiments). **(D-I)** Intracellular ROS, MDA, 12-HETE, 15-HETE, GSH and GPX activity in PG7 cells of the indicated groups treated with erastin (2 μM, 6 h), ferrostatin-1 (1 μM, 6 h), Z-VAD-FMK (10 mM, 6 h). Data indicated as mean ± S.D. (n = 3 experiments). *p < 0.05, **p < 0.01, ***p < 0.001.


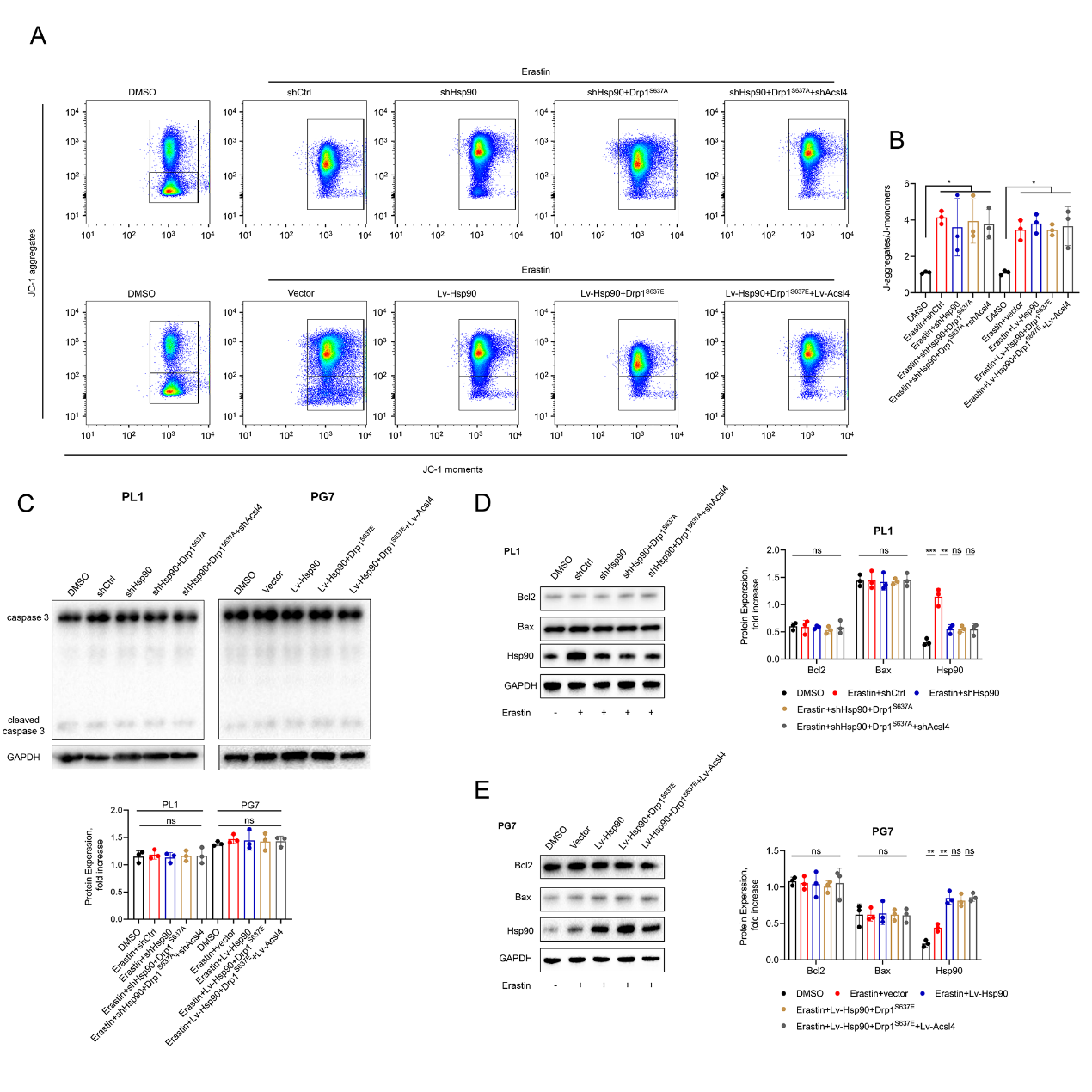


**Supplement Fig.6 Promotion of the Hsp90-Acsl4 pathway enhances Erastin sensitivity *in vitro*.**

**(A-B)** Mitochondrial membrane potential was detected by JC-1 aggregates/JC-1 monomers by flow cytometry in the indicated groups. PL1 cells were treated with 1 μM erastin and PG7 cells were treated with 2 μM erastin. Data indicated as mean ± S.D. (n = 3 experiments). **(C)** Caspase-3 level in the indicated groups in PL1 and PG7 cells as indicated treatments. **(D-E)** Expressions of Bcl2, Bax and Hsp90 were determined by western blot in the indicated groups as treated before. Data indicated as mean ± S.D. (n = 3 experiments). *p < 0.05, **p < 0.01, ***p < 0.001.


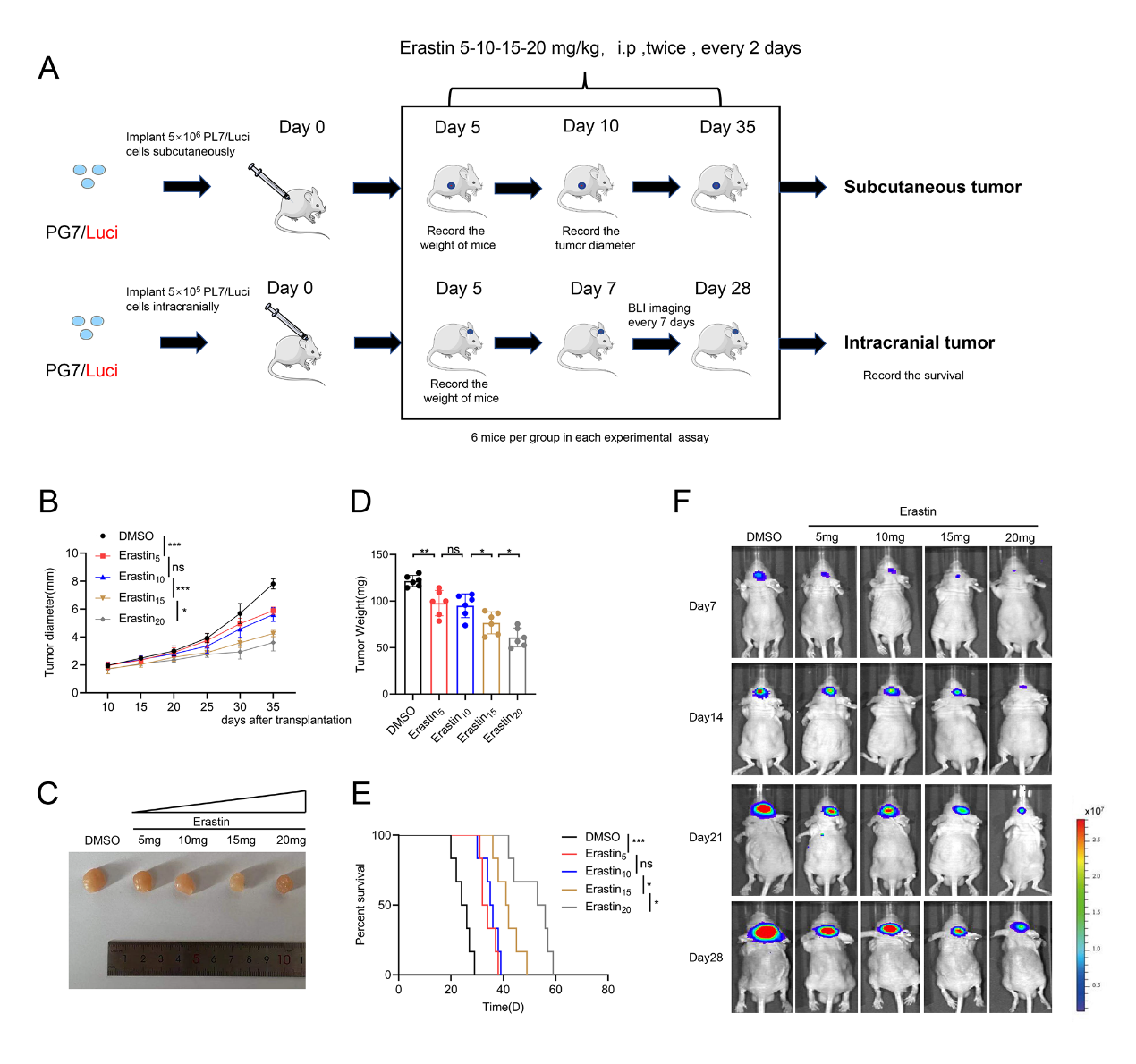


**Supplementary Fig.7 Erastin inhibits glioblastoma growth *in vivo*.**

**(A)** Mice were subcutaneously and intracranially xenografted with PG7 cells (5 × 10^6^/5 × 10^5^ cells) and treated intraperitoneally with erastin (5-10-15-20 mg kg^−1^ day^−1^ per mouse) or DMSO (0.3%) twice, every two days. **(B)** Diameter of subcutaneous tumors. Data indicated as mean ± S.D. (n = 6 mice per group). **(C)** Image of subcutaneous tumors treated with different concentrations of Erastin. **(D)** Weight of subcutaneous tumors. Data indicated as mean ± S.D. (n = 6 mice per group). **(E)** Kaplan-Meier survival of mice. (n = 6 mice per group). **(F)** Bioluminescence imaging was performed on days 7, 14, 21, and 28 after implantation. *p < 0.05, **p < 0.01, ***p < 0.001.

**Supplementary Table 1**

**Clinical information diagnosis of patients with glioma**

| **No.** | **Gender** | **Age** | **Lesion area** | **Diagnosis** | | **LGG or GBM** | |
| --- | --- | --- | --- | --- | --- | --- | --- |
| 1 | Male | 52 | Temporal Lobe | | [Oligodendroglioma](javascript:;) | LGG |  |
| 2 | Female | 29 | Basal Ganglia | | Diffuse astrocytoma | LGG |  |
| 3 | Female | 40 | Temporal and  Parietal Lobe | | [Oligodendroglioma](javascript:;) | LGG |  |
| 4 | Female | 43 | Frontal Lobe | | [Oligodendroglioma](javascript:;) | LGG |  |
| 5 | Male | 57 | Temporal and  Parietal Lobe | | [Glioblastoma](javascript:;) | GBM |  |
| 6 | Male | 32 | [Insular](javascript:;) [lobe](javascript:;) | | [Glioblastoma](javascript:;) | GBM |  |
| 7 | Female | 35 | Basal Ganglia | | [Glioblastoma](javascript:;) | GBM |  |
| 8 | Female | 44 | Frontal and Temporal Lobe | | [Glioblastoma](javascript:;) | GBM |  |
| 9 | Male | 76 | Frontal and Temporal Lobe | | [Glioblastoma](javascript:;) | GBM |  |
| 10 | Female | 60 | Frontal, Parietal and  Temporal Lobe | | [Glioblastoma](javascript:;) | GBM |  |
| 11 | Male | 47 | Temporal and  Parietal Lobe | | [Glioblastoma](javascript:;) | GBM |  |

**Supplementary Table 2**

**All candidate proteins identified by mass spectrometry**

| Protein ID | Protein Mass | PeptideSeqs | Abundance |
| --- | --- | --- | --- |
| sp\|O00429\|DNM1L_HUMAN | 81826.11108 | SQLDINNKK | 1133219.615 |
| sp\|O75152\|ZC11A_HUMAN | 89076.01151 | IDSEIK | 668586.674 |
| sp\|P07437\|TBB5_HUMAN | 49638.97066 | ISVYYNEATGGK | 119776.3407 |
| sp\|A0A0A0MRZ7\|KVD26_HUMAN | 13288.53451 | FSGSGSGTDFTLK;FSGVPDR | 114389.1976 |
| sp\|Q92766\|RREB1_HUMAN | 181306.2659 | GLLRHNALVHK | 64050.63907 |
| sp\|O15020\|SPTN2_HUMAN | 271157.1111 | DLTGALR | 60814.81374 |
| sp\|A0JNW5\|UH1BL_HUMAN | 164095.2426 | ITGVNGEIDIR | 60380.83973 |
| sp\|P07900\|HS90A_HUMAN | 84606.67999 | EDQTEYLEER;LSELLR;TLVSVTK | 57863.48885 |
| sp\|P68431\|H31_HUMAN | 15394.48309 | EIAQDFK;STELLIR | 52403.87859 |
| sp\|P04406\|G3P_HUMAN | 36030.39723 | LTGMAFR;VGVNGFGR | 49184.98174 |
| sp\|O14556\|G3PT_HUMAN | 44472.78918 | AGIALNDNFVK;LTGMAFR | 37495.91387 |
| sp\|P81605\|DCD_HUMAN | 11276.82728 | ENAGEDPGLAR | 27985.73819 |
| sp\|P62805\|H4_HUMAN | 11360.3815 | ISGLIYEETR;VFLENVIR | 27451.27041 |
| sp\|P58876\|H2B1D_HUMAN | 13927.56784 | LLLPGELAK | 27446.5447 |
| sp\|Q96HP4\|OXND1_HUMAN | 34832.20801 | VIELAVK | 26270.47594 |
| sp\|P0DOX8\|IGL1_HUMAN | 22816.08255 | VTVLGQPK | 26229.93943 |
| sp\|Q8IZQ1\|WDFY3_HUMAN | 395006.4207 | QGPSQLK | 25571.80147 |
| sp\|P0DPH8\|TBA3D_HUMAN | 49927.60194 | DVNAAIATIK;LSVDYGK | 20711.55189 |
| sp\|P0C0S5\|H2AZ_HUMAN | 13544.55174 | HLQLAIR | 18229.26591 |
| sp\|P25705\|ATPA_HUMAN | 59713.59237 | AVDSLVPIGR | 14589.43379 |
| sp\|Q9UPW5\|CBPC1_HUMAN | 138360.7958 | LPLPTIK | 13235.21237 |
| sp\|Q96HU8\|DIRA2_HUMAN | 22470.59761 | VAVFGAGGVGK | 11453.17507 |
| sp\|Q01968\|OCRL_HUMAN | 104137.9556 | QVISQLPR | 10682.43894 |
| sp\|Q16322\|KCA10_HUMAN | 57748.13791 | VIINIAGLR | 9754.945466 |
| sp\|Q5T749\|KPRP_HUMAN | 64093.23986 | LQLFPR | 9377.23605 |
| sp\|Q8N1N4\|K2C78_HUMAN | 56830.49146 | FLEQQNK;VDELEAALR | 9358.648107 |
| sp\|Q96P63\|SPB12_HUMAN | 46247.08621 | IGFIEEVK | 8180.620914 |
| sp\|P06733\|ENOA_HUMAN | 47139.3184 | IGAEVYHNLK | 7534.534457 |
| sp\|Q8WUH2\|TGFA1_HUMAN | 97095.97069 | QIQDLLASR | 7071.61364 |
