# Supplementary material for: Hsp90 induces Acsl4-dependent glioma ferroptosis via dephosphorylating Ser637 at Drp1
Source: Cell Death Dis. 2022 Jun 13;13(6):548. doi: 10.1038/s41419-022-04997-1 (PMC9192632; doi:10.1038/s41419-022-04997-1)

Figure.1E

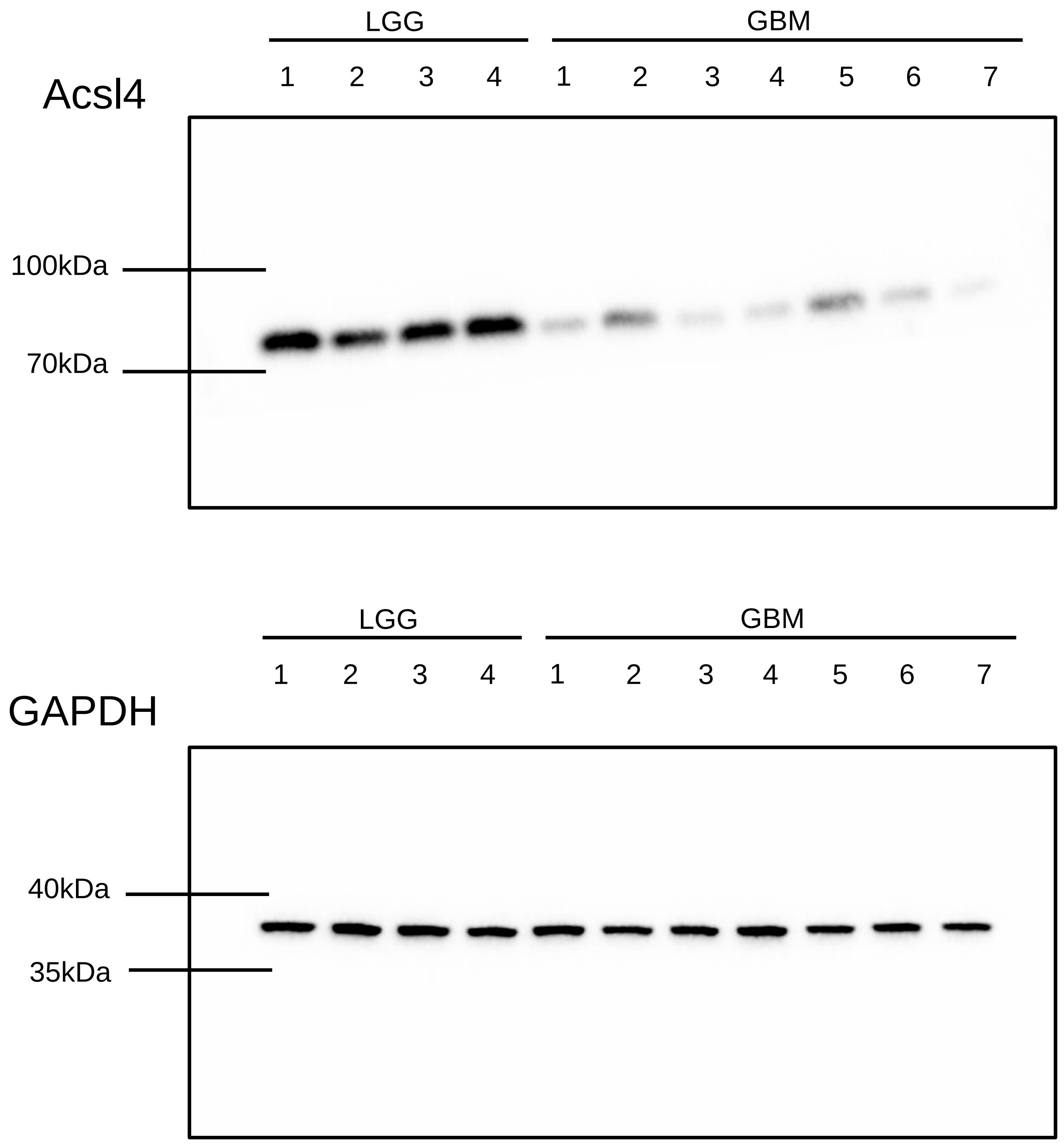
LGG
GBM
1 2 3 4 1 2 3 4 5 6 7
Acsl4
100kDa
70kDa
LGG
GBM
1 2 3 4 1 2 3 4 5 6 7
GAPDH
40kDa
35kDa

Figure.2D

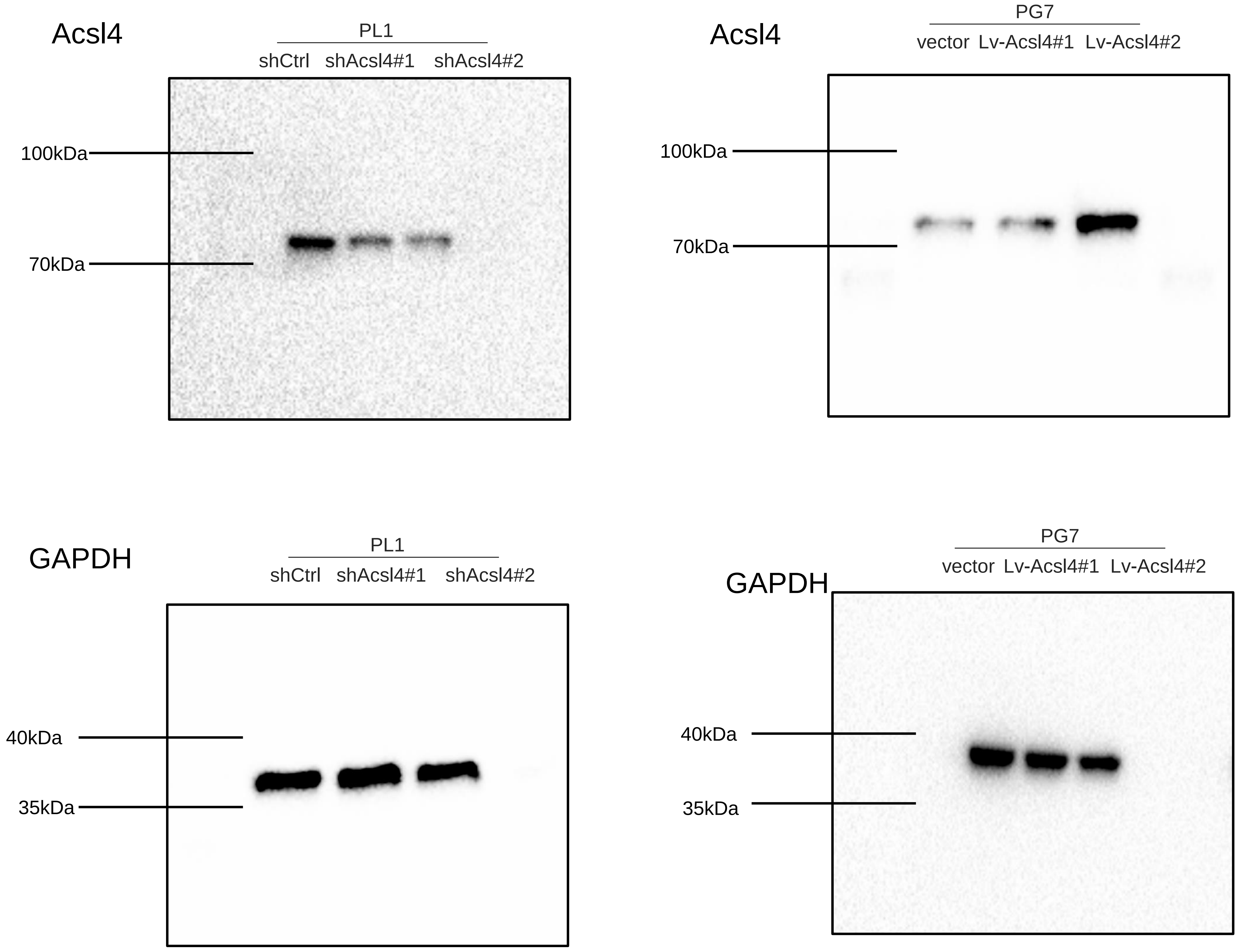
Acsl4
PL1
shCtrl shAcsl4#1 shAcsl4#2
100kDa
70kDa
Acsl4
PG7
vector Lv-Acsl4#1 Lv-Acsl4#2
100kDa
70kDa
GAPDH
PL1
shCtrl shAcsl4#1 shAcsl4#2
40kDa
35kDa
PG7
vector Lv-Acsl4#1 Lv-Acsl4#2
GAPDH
40kDa
35kDa

Figure.3D

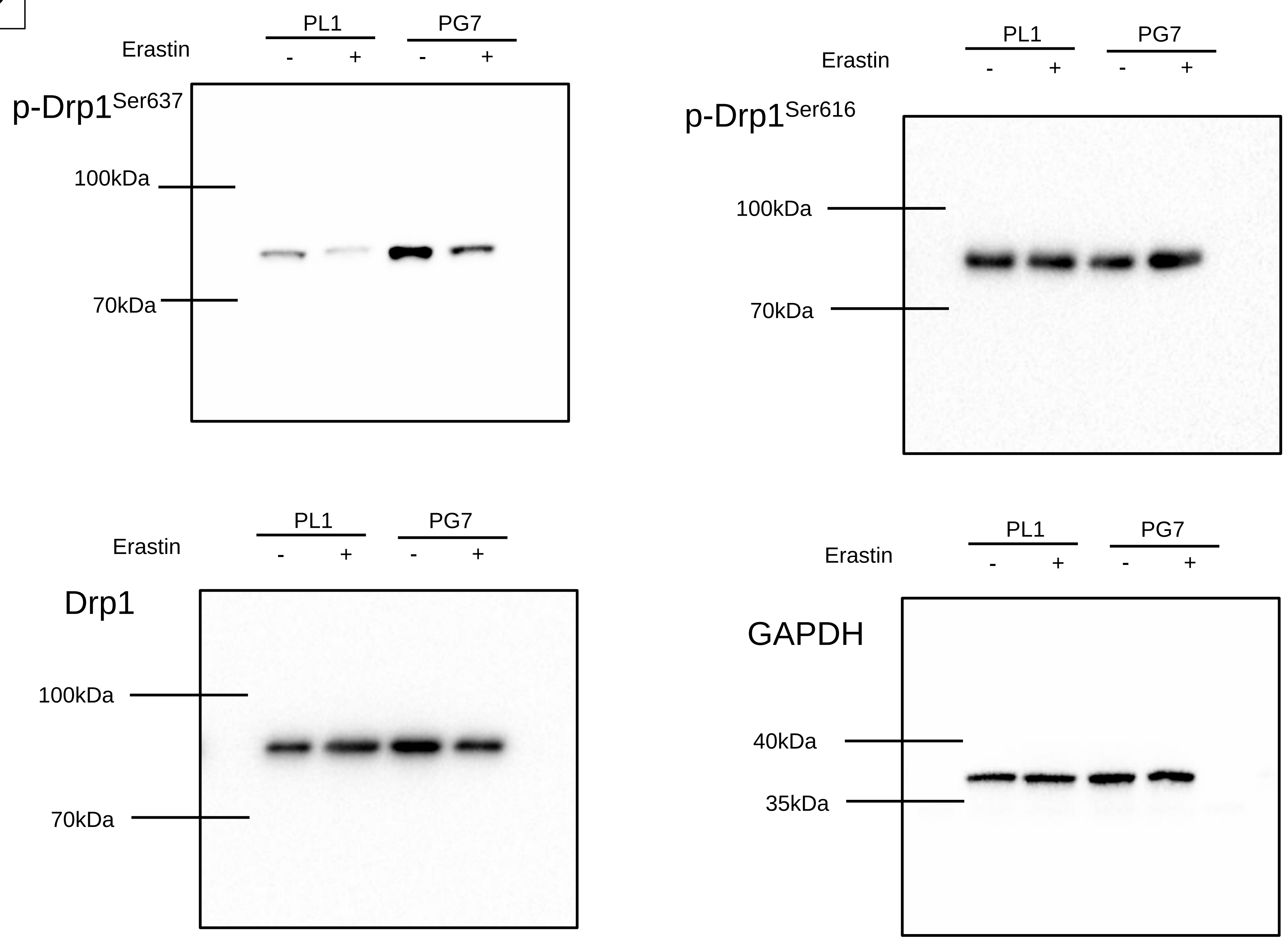

Figure.3B

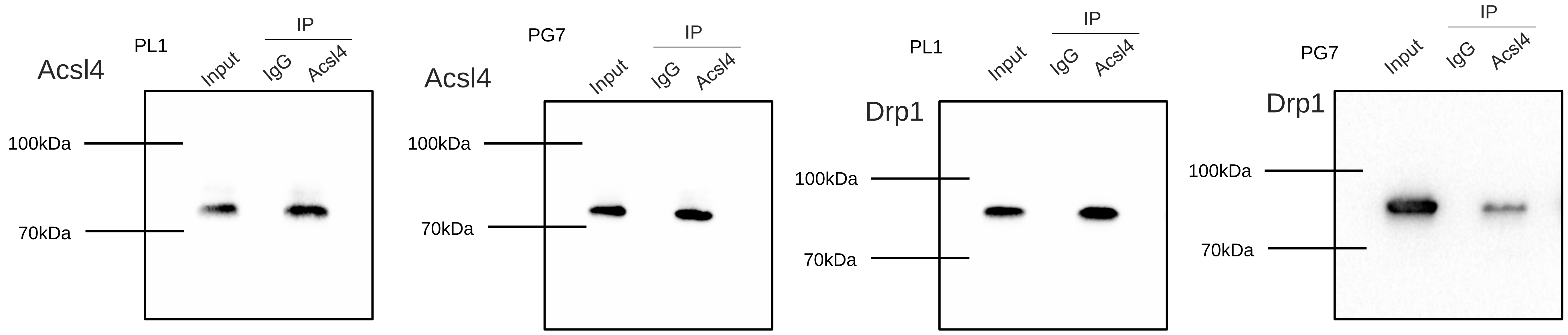

Figure.4D

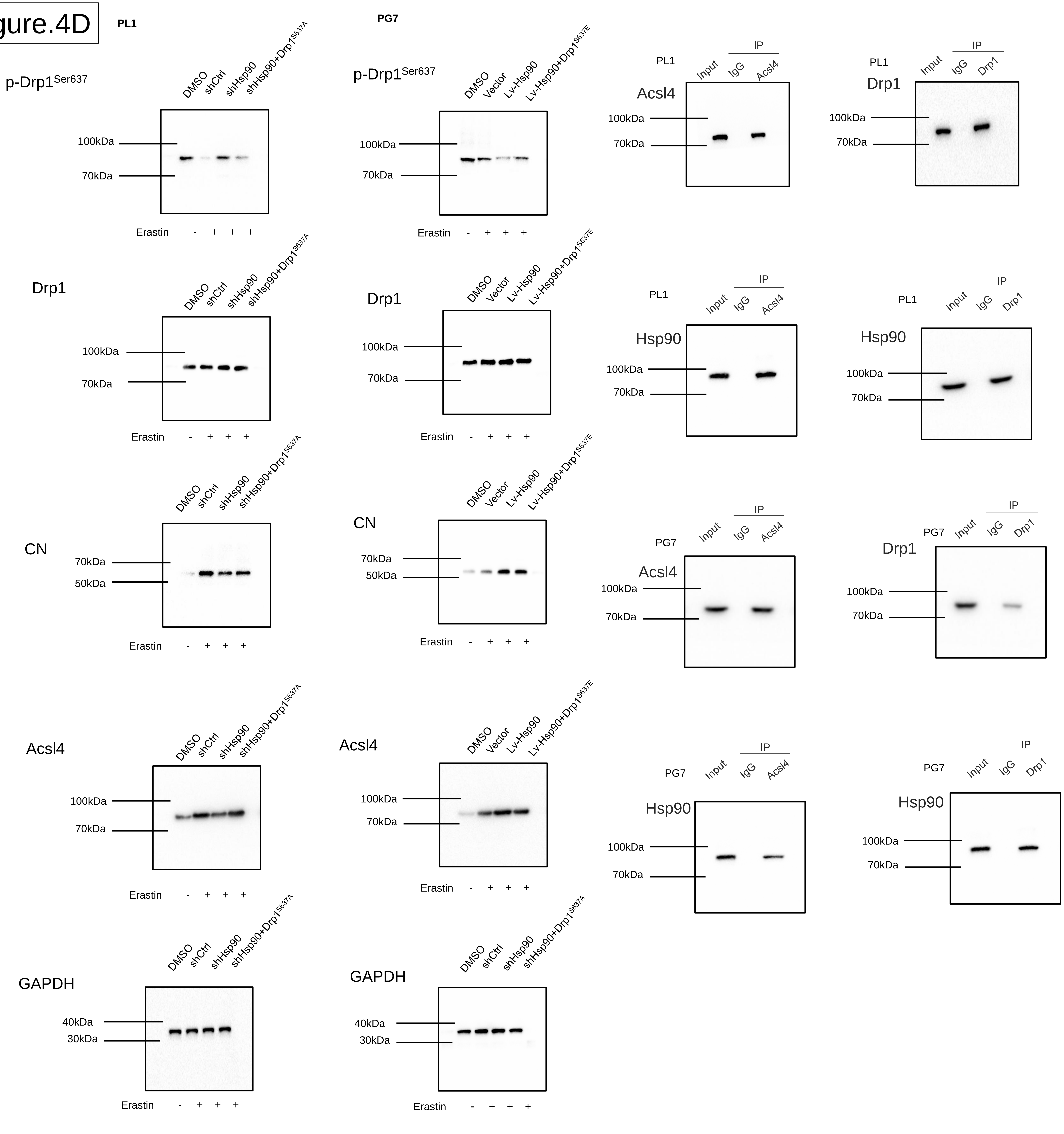

PL1
PG7
p-Drp1^{Ser637}
DMSO
shCtrl
shHsp90
shHsp90+Drp1^{S637A}
Vector
Lv-Hsp90
Lv-Hsp90+Drp1^{S637E}
100kDa
70kDa
50kDa
40kDa
30kDa
Erastin - + + +
Drp1
CN
Acsl4
GAPDH
IP
Input
IgG
Hsp90

Figure.4E,F

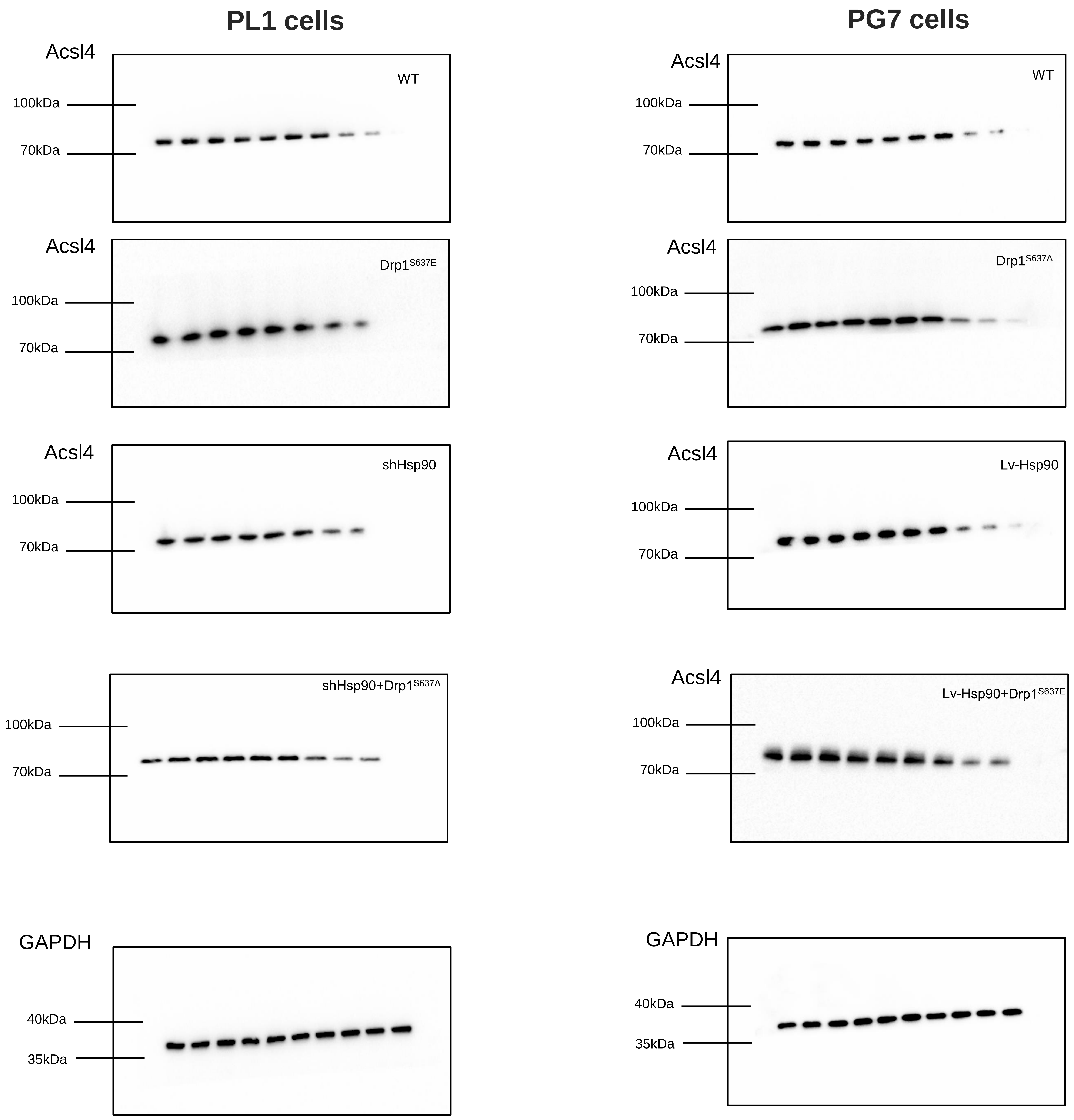
PL1 cells
PG7 cells
Acsl4
WT
100kDa
70kDa
Acsl4
Drp1S637E
100kDa
70kDa
Acsl4
shHsp90
100kDa
70kDa
shHsp90+Drp1S637A
100kDa
70kDa
GAPDH
40kDa
35kDa
Acsl4
WT
100kDa
70kDa
Acsl4
Drp1S637A
100kDa
70kDa
Acsl4
Lv-Hsp90
100kDa
70kDa
Acsl4
Lv-Hsp90+Drp1S637E
100kDa
70kDa
GAPDH
40kDa
35kDa

Figure.5A,B

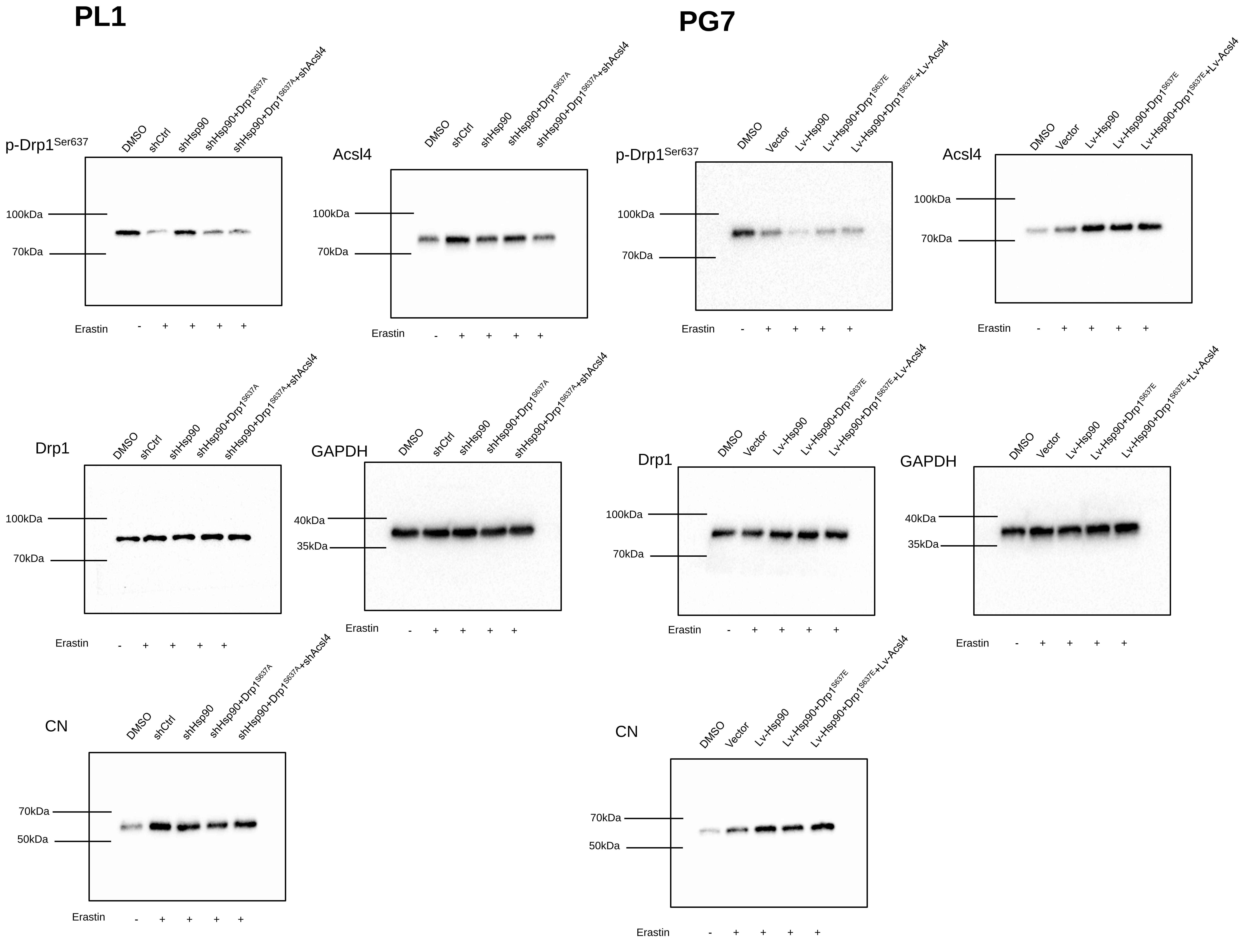

PL1
PG7
p-Drp1^{Ser637}
Acsl4
Drp1
GAPDH
CN
DMSO
shCtrl
shHsp90
shHsp90+Drp1^{S637A}
shHsp90+Drp1^{S637A}+shAcsl4
Vector
Lv-Hsp90
Lv-Hsp90+Drp1^{S637E}
Lv-Hsp90+Drp1^{S637E}+Lv-Acsl4
100kDa
70kDa
50kDa
40kDa
35kDa
Erastin
- + + + +

Supplement Fig.1A, C

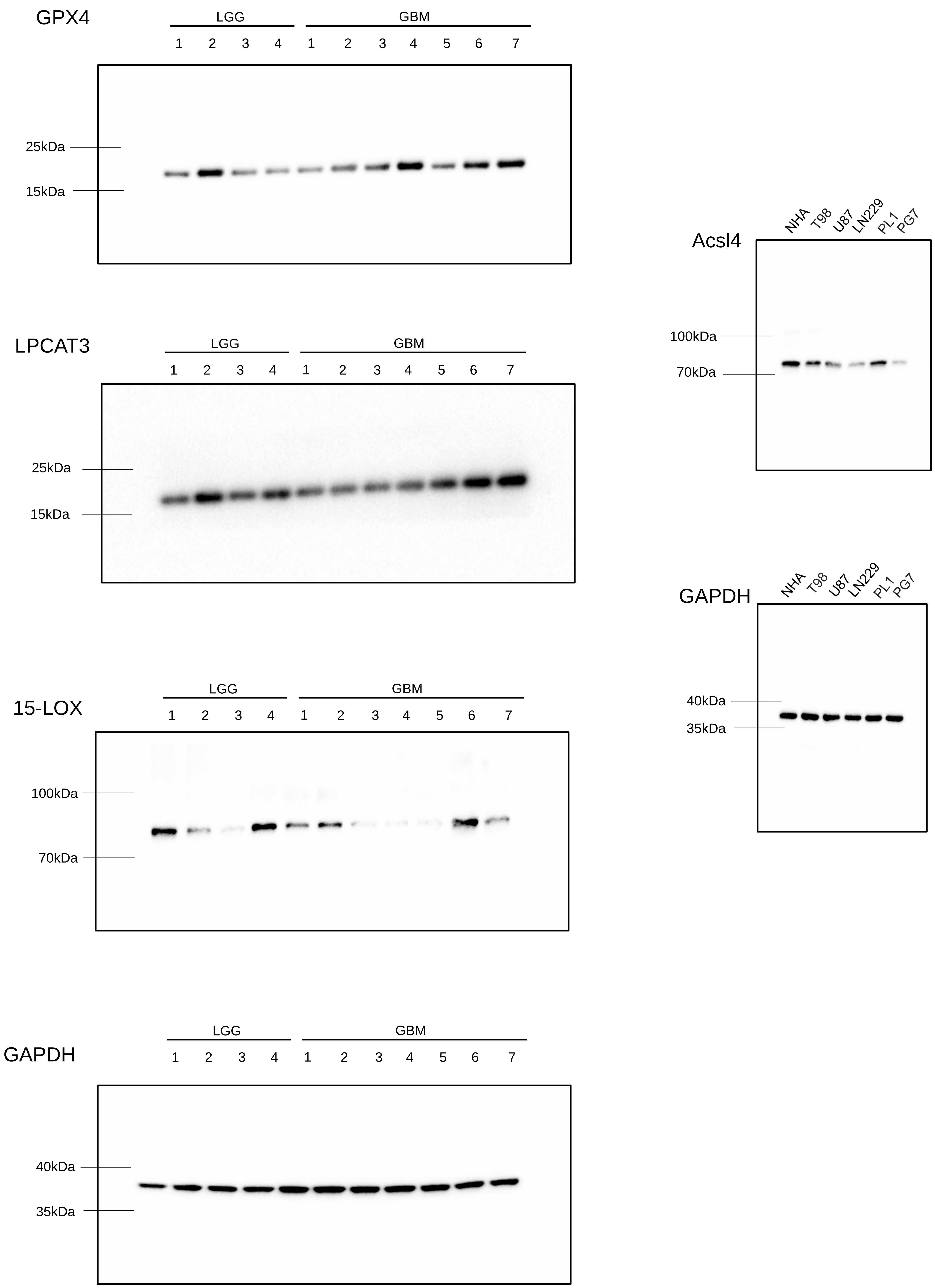

Supplement Fig.2A, C

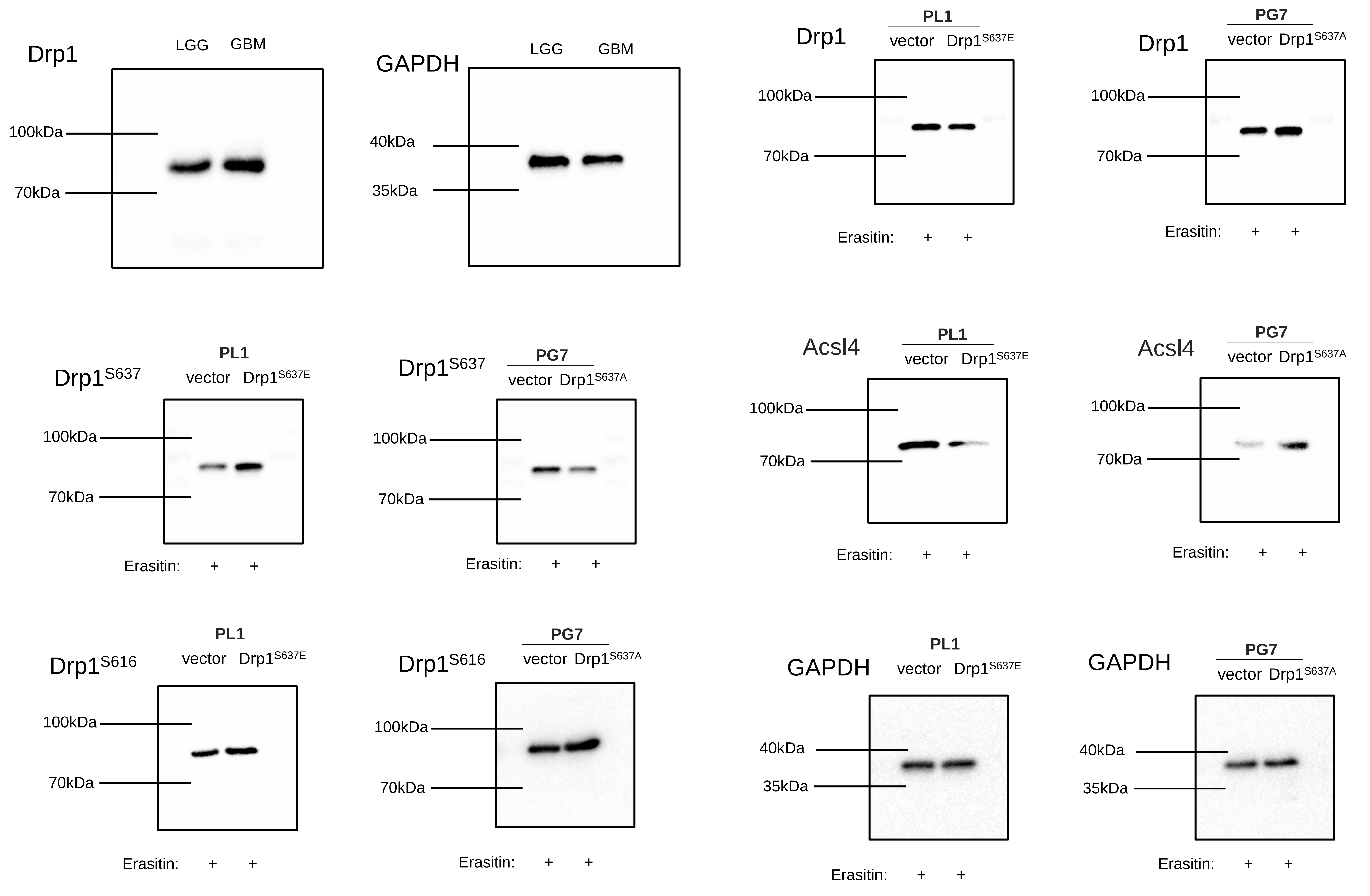

Supplement Fig.4A, B, D

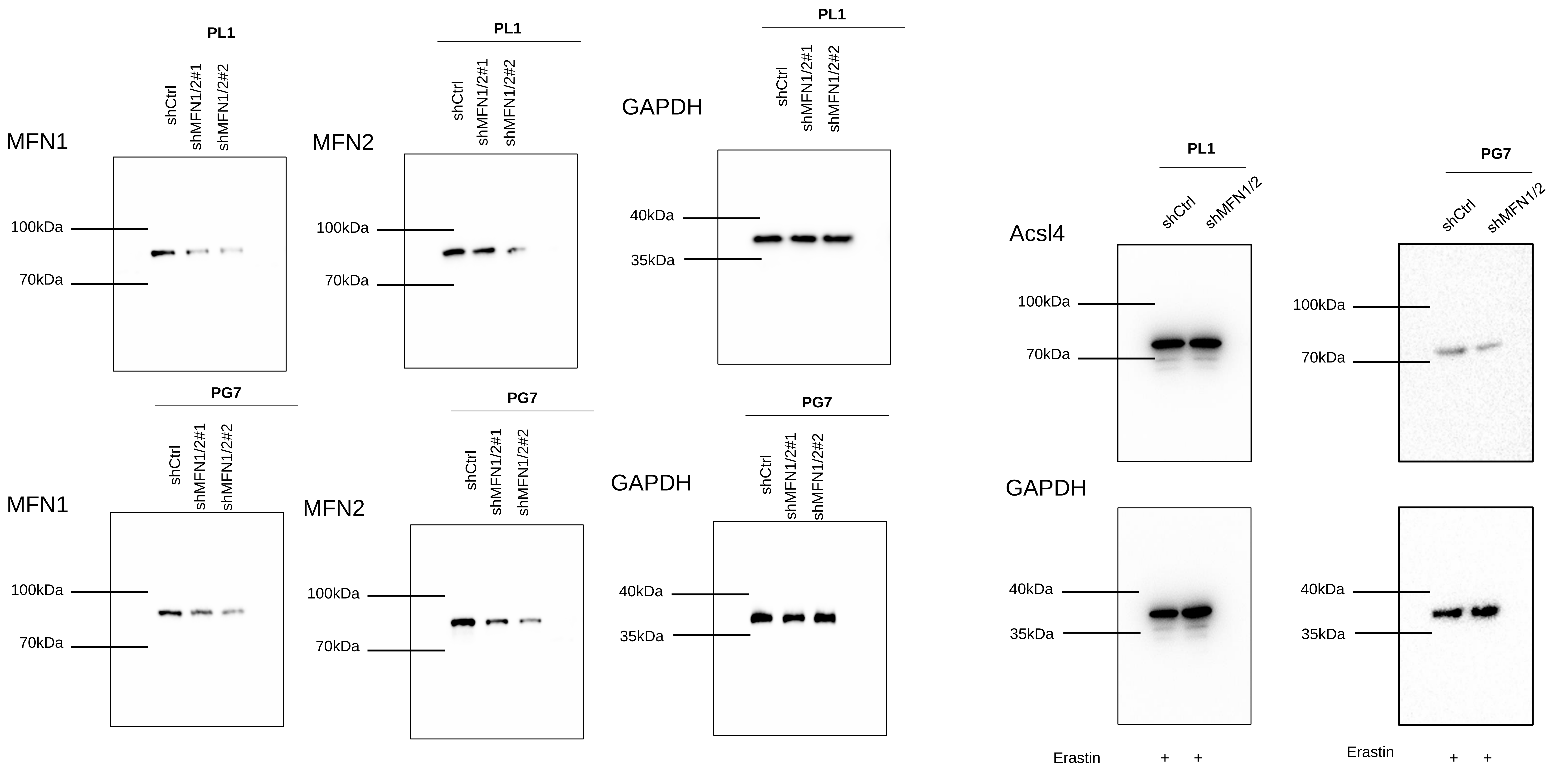

Supplement Fig.5C

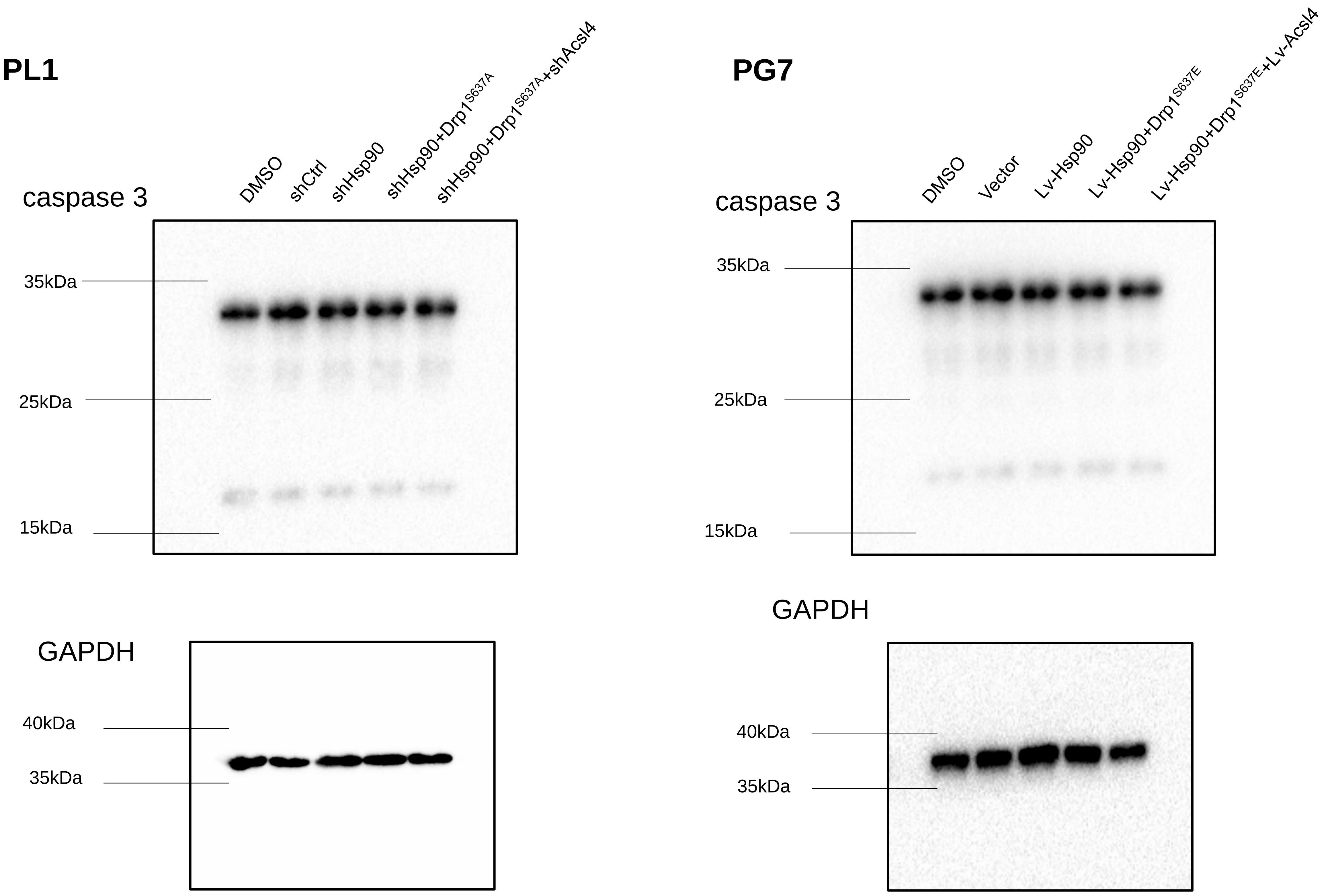

Supplement Fig.5D

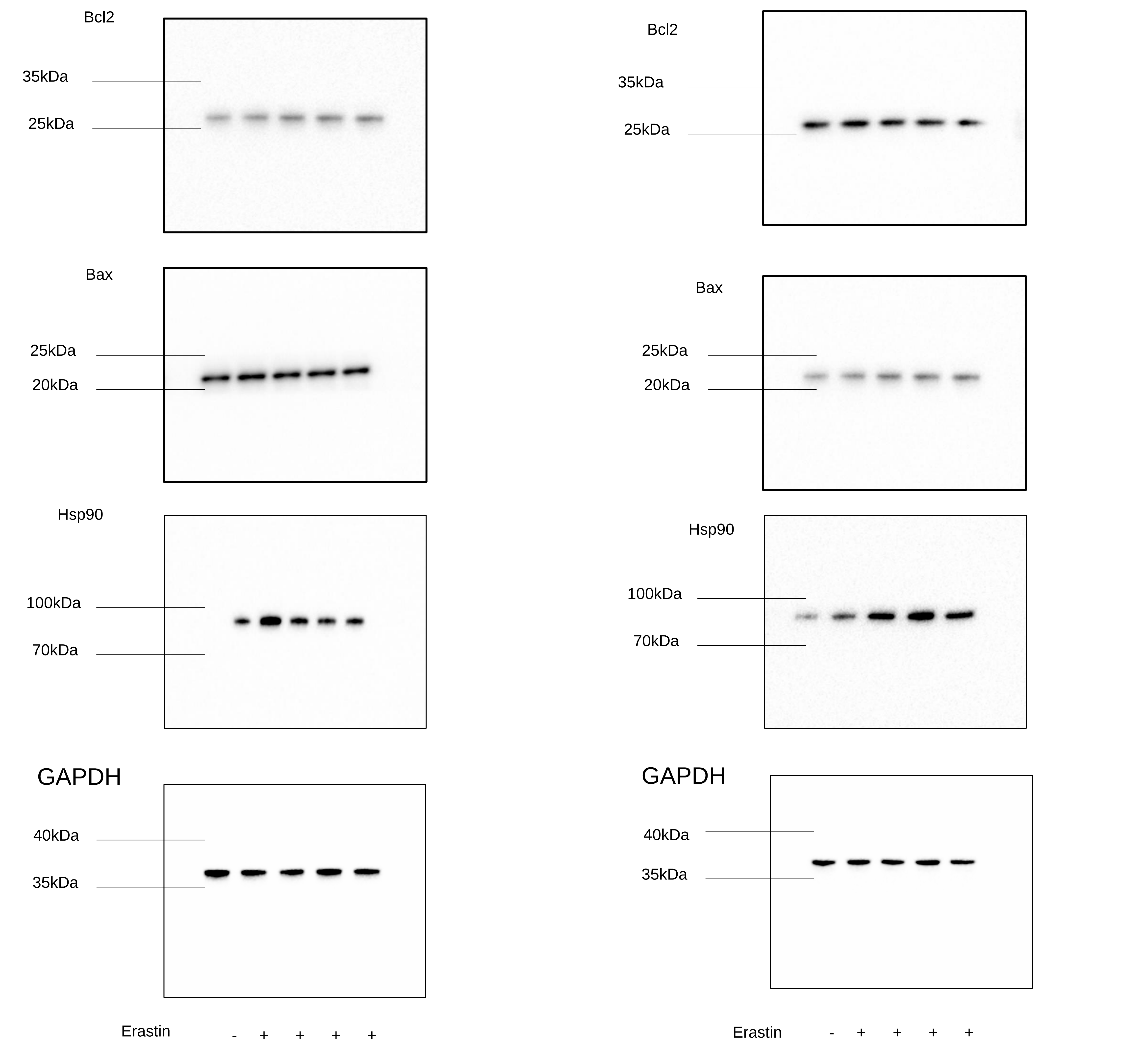

rebuttal letter1

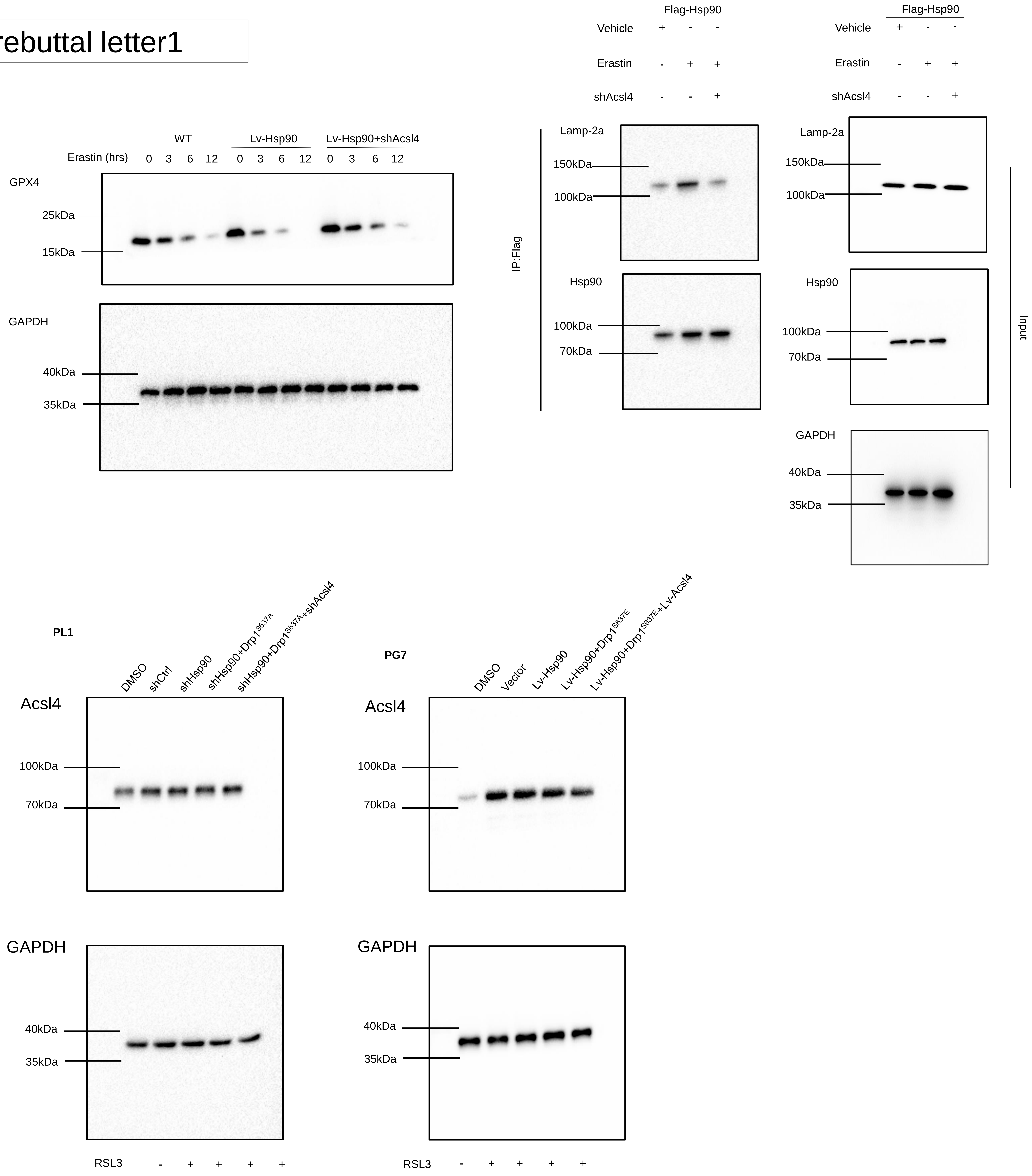

rebuttal letter 2 Q1

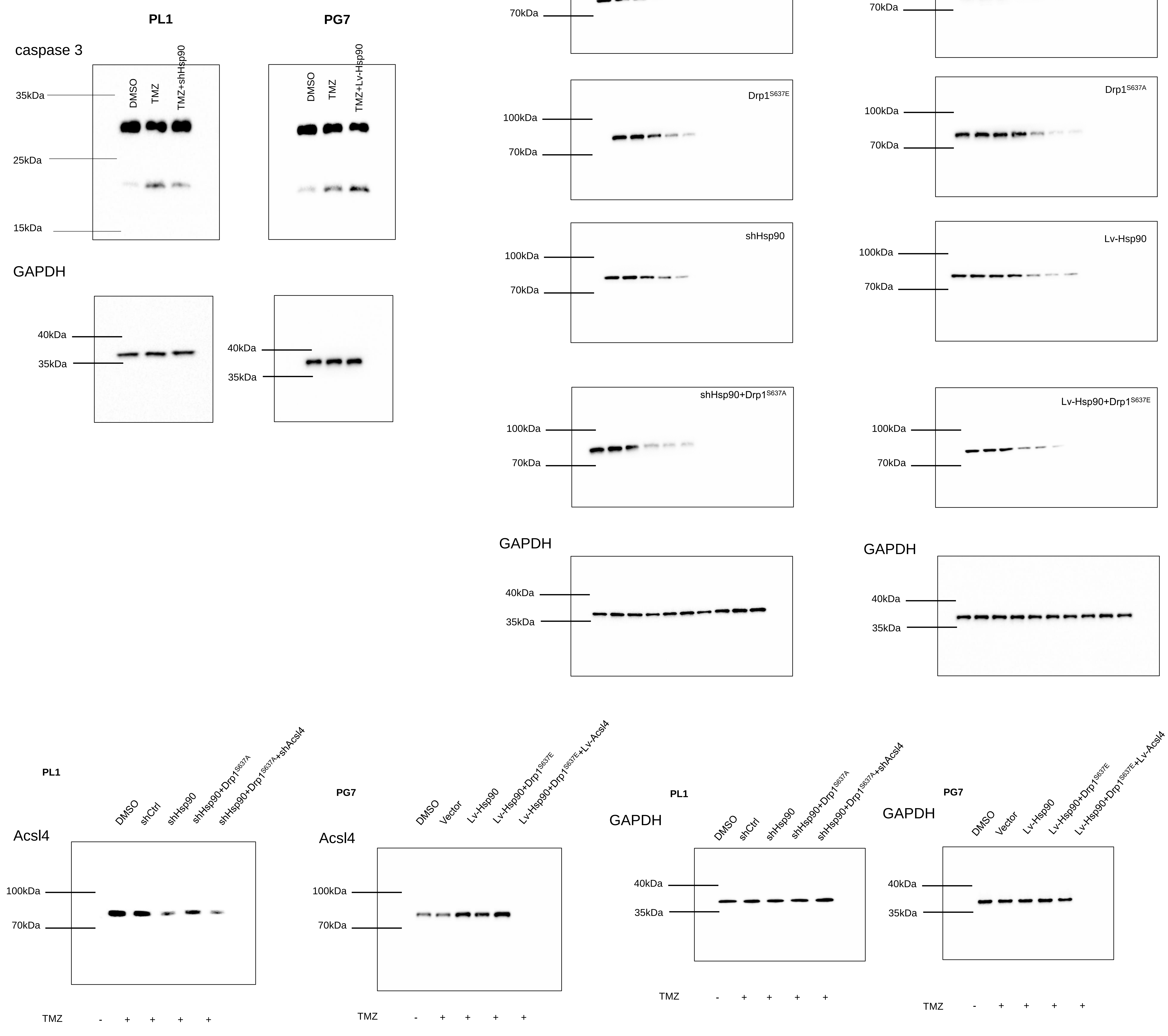

rebuttal letter Q5

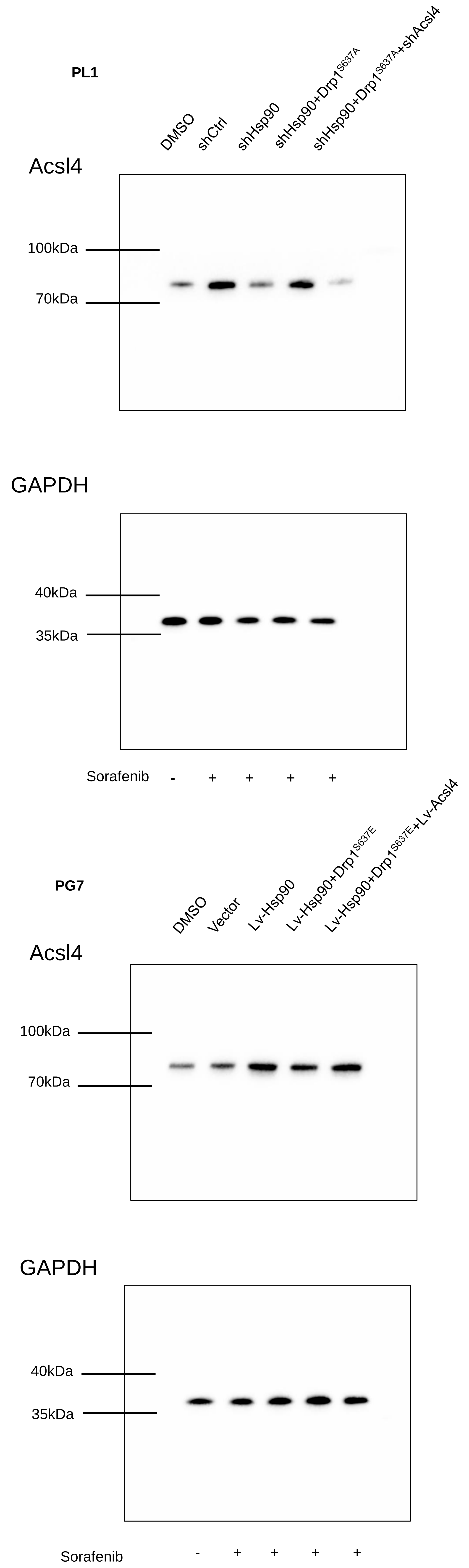

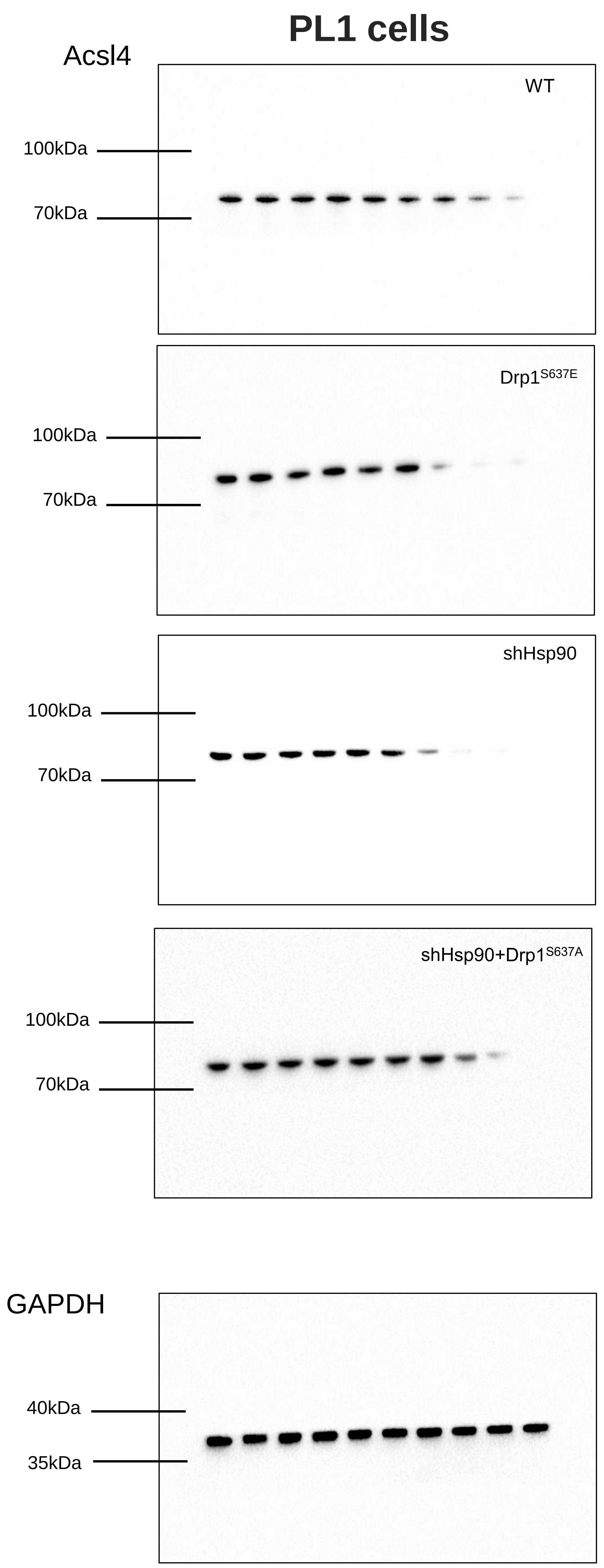

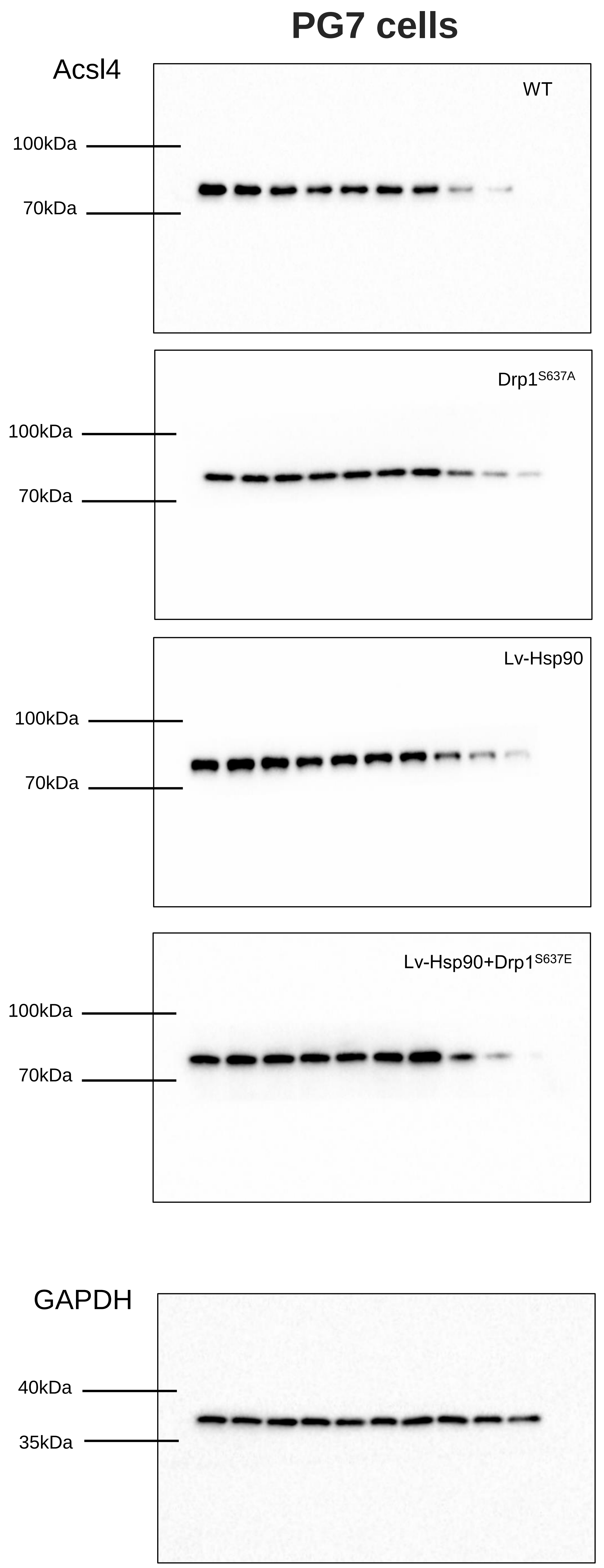

Supplement: Supplementary file 2 — original western blots [file 41419_2022_4997_MOESM2_ESM.pdf]
